# Efficacy and safety of tirzepatide for weight loss in patients with obesity or type 2 diabetes: a systematic review and meta-analysis

**DOI:** 10.3389/fendo.2025.1593134

**Published:** 2025-07-17

**Authors:** Qiru Tian, Yi Song, Yan Deng, Shike Lin

**Affiliations:** ^1^ Hainan Vocational University of Science and Technology, Haikou, China; ^2^ Faculty of Medicine, Chinese University of Hong Kong, Hong Kong, Hong Kong SAR, China; ^3^ Office of Science and Technology, Youjiang Medical University for Nationalities, Baise, Guangxi, China; ^4^ Faculty of Nursing, Youjiang Medical University for Nationalities, Baise, Guangxi, China; ^5^ Department of Obstetrics and Gynaecology, Affiliated Hospital of Youjiang Medical University for Nationalities, Baise, Guangxi, China

**Keywords:** tirzepatide, weight loss, meta-analysis, obesity, type 2 diabetes, adverse events

## Abstract

**Background:**

This meta-analysis aims to evaluate efficacy and safety of tirzepatide for weight loss, including its dose-response relationship and adverse event profile.

**Methods:**

Studies were retrieved from high-impact journals and included phase 1 to phase 3 trials. Participants received tirzepatide at 5,10, or 15 mg doses or a placebo control. Weighted mean differences (WMD) and odds ratios (OR) with 95% confidence intervals (CIs) were used to evaluate treatment effects, and heterogeneity was assessed using I² statistic.

**Results:**

Tirzepatide induced a mean weight reduction of –10.39 kg versus placebo (95% CI: –10.80 to –9.99; p < 0.00001). Subgroup analyses by diabetes status showed that patients with type 2 diabetes lost –6.17 kg (95% CI: –7.16 to –5.17; p < 0.00001) at 5 mg, –8.57 kg (95% CI: –9.41 to –7.74; p < 0.00001) at 10 mg, and –9.60 kg (95% CI: –10.32 to –8.89; p < 0.00001) at 15 mg. Non-diabetic participants experienced greater absolute losses of –12.10 kg (95% CI: –13.47 to –10.72; p < 0.00001), –15.94 kg (95% CI: –17.25 to –14.62; p < 0.00001), and –17.86 kg (95% CI: –19.19 to –16.54; p < 0.00001) at the respective doses. Tirzepatide also markedly increased the odds of achieving clinically meaningful weight loss: ≥ 5% (OR=11.32; p < 0.0001), ≥ 10% (OR=14.77; p < 0.0001), and ≥ 15% (OR=18.07; p < 0.0001. Adverse events were more frequent with tirzepatide than placebo (OR=1.34; p < 0.0001), largely driven by gastrointestinal symptoms, whereas serious adverse events did not differ. Discontinuations due to side effects increased at higher doses (OR=2.31; p < 0.0001).

**Conclusions:**

Tirzepatide induces significant, dose-dependent weight loss, with higher doses yielding greater reductions. While gastrointestinal side effects were common, they were generally mild to moderate and did not increase serious adverse events. These findings support tirzepatide as an effective weight management therapy, though strategies to mitigate gastrointestinal symptoms may improve adherence.

## Introduction

Diabetes mellitus and obesity are two interrelated global health challenges that significantly contribute to morbidity, mortality, and healthcare expenditures (1). Type 2 diabetes mellitus (T2DM) is featured by beta-cell malfunction and insulin resistance (2, 3), while obesity exacerbates metabolic dysfunction and potentiates risk of developing T2DM and other chronic diseases (4). Traditional pharmacologic interventions have shown limited long-term success in maintaining glycemic control and reducing weight, necessitating the development of novel therapeutic agents with dual efficacy (5, 6). Tirzepatide, a novel dual glucagon-like peptide-1 (GLP-1) and glucose-dependent insulinotropic polypeptide (GIP) receptor agonist, has gained recognition as an effective option for managing both T2DM and obesity by targeting multiple metabolic pathways (5–8).

Clinical trials have increasingly confirmed that tirzepatide induces reductions in glycated hemoglobin (HbA1c) levels and body weight comparing with conventional antidiabetic and anti-obesity treatments (9–13). Its unique mechanism of action, which boosts insulin output while simultaneously curtails glucagon production and reducing appetite, suggests a potential paradigm shift in the management of metabolic disorders (14–16). However, while tirzepatide has shown superior efficacy, concerns remain regarding its long-term safety profile, particularly with respect to gastrointestinal adverse effects, pancreatitis, and cardiovascular outcomes (8, 17). A comprehensive evaluation of its benefits and risks is necessary to assist in clinical choices and influence upcoming therapeutic strategies.

Meta-analyses are essential for integrating findings from clinical trials to provide robust assessment of drug safety and efficacy. Previous meta-analyses have evaluated GLP-1 receptor agonists treating obesity and diabetes, but specific impact of dual GLP-1/GIP receptor agonism remains insufficiently explored (8, 18–22). Given the growing interest in tirzepatide as a first-line or adjunctive therapy, an updated and comprehensive synthesis of the available evidence is warranted. Examining both glycemic and weight-related outcomes, along with adverse events, will provide clinicians and policymakers with a clearer understanding of tirzepatide’s clinical utility.

Therefore, a comprehensive review of randomized controlled trials (RCTs), our meta-analysis investigate safety and efficacy of tirzepatide in patients with diabetes or obesity. Our primary objectives include assessing its impact on weight loss while also investigating adverse events and tolerability. By integrating the latest available data, our works seeks to offer research-backed findings into roles of tirzepatide for managing metabolic dysfunctions, contributing to its optimal use in clinical practice.

## Methods

### Study design and eligibility criteria

Following the PRISMA guidelines, this meta-analysis was systematically conducted. RCTs investigating tirzepatide’s effectiveness and safety in patients with T2DM or obesity were considered for inclusion. Studies were eligible if they (1) compared tirzepatide to placebo, (2) reported at least one efficacy outcome (e.g., change in glycated hemoglobin [HbA1c] levels or body weight) or one safety outcome (e.g., incidence of adverse events), (3) had a follow-up duration of at least 8 weeks, and (4) were published in peer-reviewed journals. Non-human studies, trials involving pediatric participants, and non-randomized designs were not included.

### Search strategy

Relevant studies were systematically searched in PubMed, Embase, Cochrane CENTRAL and Web of Science from inception to March 1st, 2025. The search methodology combined MeSH terms and specific keywords associated with ‘tirzepatide,’ ‘diabetes mellitus,’ ‘obesity,’ ‘randomized controlled trial,’ ‘efficacy,’ and ‘safety.’ Additionally, relevant articles were manually retrieved from reference lists and conference proceedings, with no restrictions on language.

### Study selection and data extraction

Titles and abstracts were screened independently by two reviewers, with full-text assessments conducted for eligible studies. Conflicts were addressed through discussion or adjudication by a third reviewer. Data extraction followed a standardized template, collecting information on study characteristics (authorship, publication year, sample size, design and study duration), population characteristics (age, sex, baseline HbA1c, BMI), intervention details (tirzepatide dosage, comparator, treatment duration), primary and secondary outcomes, and safety data. In cases of missing information, the corresponding authors were approached for clarification.

### Risk of bias assessment

To evaluate study quality, the Cochrane risk of bias tool was applied, assessing seven methodological domains: randomization, allocation concealment, participant and personnel blinding, blinding of outcome assessment, handling of missing data, selective reporting, and other biases. Each domain was categorized as having a low, high, or unclear risk of bias.

### Statistical analysis

Meta-analysis was carried out using Review Manager (RevMan), applying a random-effects model to address between-study heterogeneity. Weighted mean differences (WMD) with 95% confidence intervals (CI) were used to analyze continuous outcomes, such as body weight changes and body weight loss, while odds ratios (OR) with 95% CI were utilized for dichotomous outcomes, including adverse events. Study heterogeneity was quantified using the I² statistic, with thresholds of > 50% and P <0.05 representing heterogeneity. Funnel plots were examined for potential publication bias, and subgroup analyses were performed based on baseline tirzepatide dose.

## Results

### Study flow and selection of studies

**Figure 1:**
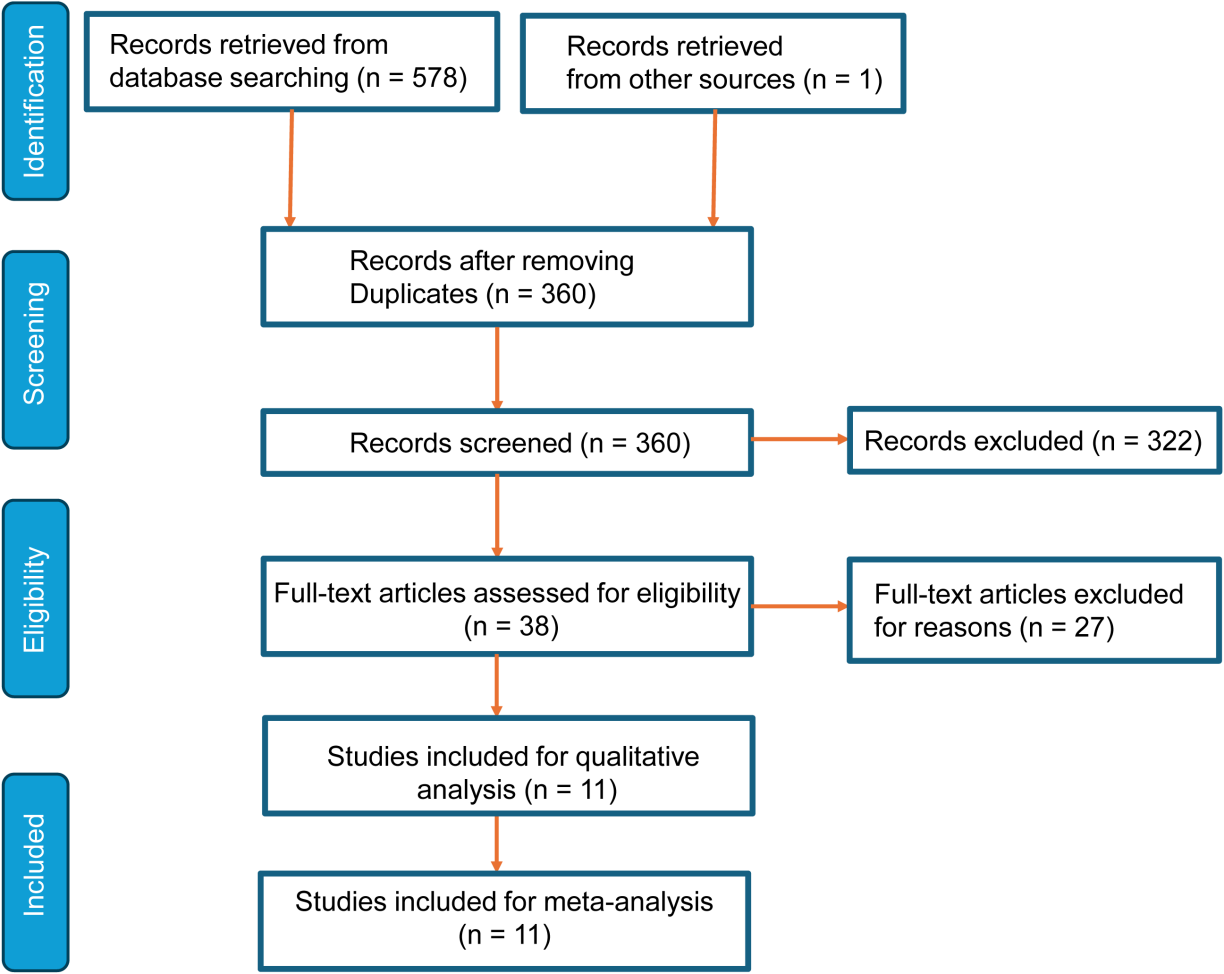
Study flow of meta-analysis.

As shown in **Figure 1**, a comprehensive search yielded 578 records from databases (n=578) and other sources (n=1). After removing duplicates, 358 unique records were screened. A total of 360 records were excluded based on title and abstract evaluation due to irrelevance or failure to meet the eligibility criteria. Of the 38 full-text articles assessed, 27 were excluded due to reasons such as incomplete data or unsuitable study design. Finally, eleven studies met inclusion criteria and were incorporated into qualitative synthesis and meta-analysis, ensuring a rigorous and relevant data selection process.

### Study characteristics

The study characteristics were summarized in **Table 1**. Our meta-analysis included ten studies assessing the effects of tirzepatide on body weight reduction. The studies were conducted across various geographical locations, including multi-country trials, as well as country-specific studies from China, Japan, Germany, and the United States. The included trials were published in high-impact journals, indicating the robustness of the evidence base. The studies encompassed phase 1 to phase 3 clinical trials, with therapy durations ranging from 8 to 176 weeks. Across all trials, participants received either tirzepatide (5, 10, or 15 mg) or a placebo. Participants’ age ranged from 34.7 to 62 years, with varying proportions of male participants (ranging from 32.2% to 100%). Baseline characteristics indicated that participants generally had a BMI range of 22.6 to 38.2 kg/m² and a body weight between 63.0 and 105.8 kg, depending on the study population and inclusion criteria. Most studies included patients with elevated HbA1c levels (ranging from 5.6% to 8.5%), reflecting populations with type 2 diabetes or obesity-related metabolic conditions. Overall, the included studies represent a diverse range of patient populations, trial designs, and geographical regions, providing a comprehensive evaluation of tirzepatide’s effects on body weight reduction across different doses and treatment durations.

**Table 1:** Study characteristics of the included studies.

Study and year	Study site	Published journal	Study design	Study groups	Patient (n)	Mean age (years old)	Gender male n (%)	Body weight (kg)	BMI (kg/m2)	HbA1c (%)	Therapy duration (weeks)
Dahl et al., 2022 (23)	48 sites in 8 countries	JAMA	SURPASS-5, Phase 3	Placebo	120	60 ± 10	66 (55%)	94.1 ± 21.8	33.2 ± 6.3	8.37 ± 0.84	40
				5 mg TZP	116	62 ± 10	61 (53%)	95.8 ± 19.8	33.4 ± 6.2	8.3 ± 0.88	
				10 mg TZP	119	60 ± 10	72 (61%)	94.5 ± 22.2	33.4 ± 5.9	8.36 ± 0.83	
				15 mg TZP	120	61 ± 10	65 (54%) 6	96.3 ± 22.8	33.2 ± 6.3	8.23 ± 0.86	
Feng et al., 2023 (24)	2 sites in China	Advances in Therapy	Phase 1	Placebo	4	56.5 ± 7.5	3 (75%)	71.3 ± 7.1	26.4 ± 1.8	7.8 ± 0.7	24
				10 mg TZP	10	55.8 ± 5.2	4 (40%)	65.0 ± 8.5	26.0 ± 2.9	8.2 ± 1.2	
				15 mg TZP	10	56.8 ± 5.4	6 (60%)	67.6 ± 6.8	25.1 ± 1.6	7.7 ± 1.1	
Frias et al., 2018 (25)	47 sites in 4 countries	Lancet	Phase 2b	Placebo	51	56.6 ± 8.9	29 (57%)	91.5 ± 23.1	32.4 ± 6.0	8.0 ± 0.9	26
				5 mg TZP	55	57.9 ± 8.2	34 (62%)	92.8 ± 19.0	32.9 ± 5.7	8.2 ± 1.0	
				10 mg TZP	51	56.5 ± 9.9	30 (59%)	92.7 ± 19.5	32.6 ± 5.8	8.2 ± 1.1	
				15 mg TZP	53	56.0 ± 7.6	22 (42%)	89.1 ± 22.7	32.2 ± 6.2	8.1 ± 1.1	
Frias et al., 2020 (26)	13 sites in United States	Diabetes, Obesityand Metabolism	Phase 2	Placebo	26	56.0 ± 10.13	12 (46.2%)	89.6 ± 23.70	32.5 ± 5.70	8.2 ± 1.22	12
				15 mg-1 TZP	28	55.5 ± 8.54	16 (57.1%)	88.7 ± 18.21	32.0 ± 5.56	8.5 ± 1.17	
				15 mg-2 TZP	28	56.6 ± 9.21	23 (82.1%)	89.6 ± 16.91	31.1 ± 4.21	8.4 ± 1.12	
Furihata et al., 2021 (27)	2 sites in Japan	Diabetes, Obesity and Metabolism	Phase 1	Placebo	9	57.4 ± 11.6	9 (100%)	63.0 ± 7.8	22.6 ± 2.1	7.8 ± 0.9	8
				5 mg TZP	11	57.5 ± 7.9	11 (100%)	75.4 ± 11.0	26.7 ± 3.3	7.7 ± 0.3	
				10 mg TZP	12	56.9 ± 9.5	12 (100%)	74.9 ± 9.5	25.5 ± 2.8	8.1 ± 0.8	
				15 mg TZP	16	57.7 ± 8.	15 (93.8%)	73.3 ± 9.9	26.1 ± 3.1	8.2 ± 0.9	
Garvey et al., 2023 (10)		Lancet	SURMOUNT-2, Phase 3	Placebo	315	54.7 ± 10.5	156 (50%)	101.7 ± 22.3	36.6 ± 7.3	7.89 ± 0.84	72
				10 mg TZP	312	54.3 ± 10.7	154 (49%)	100.9 ± 20.9	36.0 ± 6.4	8.00 ± 0.84	
				15 mg TZP	311	53.6 ± 10.6	152 (49%)	99.6 ± 20.1	35.7 ± 6.1	8.07 ± 0.99	
Heise et al., 2022 (28)	2 sites in Germany	Lancet	Phase 1	Placebo	28	60.4 ± 7.6	21 (75%)	98.74 ± 14.61	32.24 ± 3.96	7.90 ± 0.51	28
				15 mg TZP	45	61.1 ± 7.1	31 (69%)	94.15 ± 13.99	31.28 ± 5.01	7.83 ± 0.72	
Jastreboff et al., 2022 (11)	119 sites in 9 countries	New England Journal of Medicine	SURMOUNT-1, Phase 3	Placebo	643	44.4 ± 12.5	207 (32.2%)	104.8 ± 21.37	38.2 ± 6.89	5.6 ± 0.38	72
				5 mg TZP	630	45.6 ± 12.7	204 (32.4%)	102.9 ± 20.71	37.4 ± 6.63	5.6 ± 0.36	
				10 mg TZP	636	44.7 ± 12.4	209 (32.9%)	105.8 ± 23.32	38.2 ± 7.01	5.6 ± 0.37	
				15 mg TZP	630	44.9 ± 12.3	205 (32.5%)	105.6 ± 22.92	38.1 ± 6.69	5.6 ± 0.41	
Rosenstock et al., 2021 (12)	52 sites in 4 countries	Lancet	SURPASS-1, Phase 3	Placebo	115	53.6 ± 12.8	56 (49%)	84.8 ± 20.0	31.7 ± 6.1	8.05 ± 0.80	40
				5 mg TZP	121	54.1 ± 11.9	56 (46%)	87.0 ± 21.2	32.2 ± 7.0	7.97 ± 0.84	
				10 mg TZP	121	55.8 ± 10.4	72 (60%)	86.2 ± 19.5	32.2 ± 7.6	7.90 ± 0.78	
				15 mg TZP	121	52.9 ± 12.3	63 (52%)	85.4 ± 18.5	31.5 ± 5.5	7.85 ± 1.02	
Jastreboff et al., 2025 (29)	118 sites in 9 countries	New England Journal of Medicine	SURMOUNT-1, Phase 3	Placebo	270	47.7 ± 11.9	100 (37%)	107.3 ± 21.97	39.1 ± 7.10	5.77 ± 0.33	176
				5 mg TZP	247	49.3 ± 12.2	87 (35.2%)	104.6 ± 21.91	37.8 ± 6.63	5.79 ± 0.30	
				10 mg TZP	262	47.4 ± 11.6	94 (35.9%)	108.9 ± 23.88	39.0 ± 7.15	5.74 ± 0.33	
				15 mg TZP	253	48.4 ± 11.7	92 (36.4%)	108.6 ± 25.44	39.2 ± 7.43	5.76 ± 0.39	
Zhao et al., 2024 (30)	29 sites in China	JAMA	SURMOUNT-CN, Phase 3	Placebo	69	37.8 ± 10.2	36 (52.2%)	92.0 ± 15.8	32.4 ± 3.6	5.65 ± 0.29	52
				10 mg TZP	70	34.7 ± 7.2	35 (50.0%)	92.2 ± 16.2	32.6 ± 4.1	5.57 ± 0.32	
				15 mg TZP	71	35.8 ± 9.3	36 (50.7%)	91.3 ± 16.2	32.0 ± 3.7	5.60 ± 0.35	

TZP, tirzepatide.

### Risk of bias assessment

**Figure 2:**
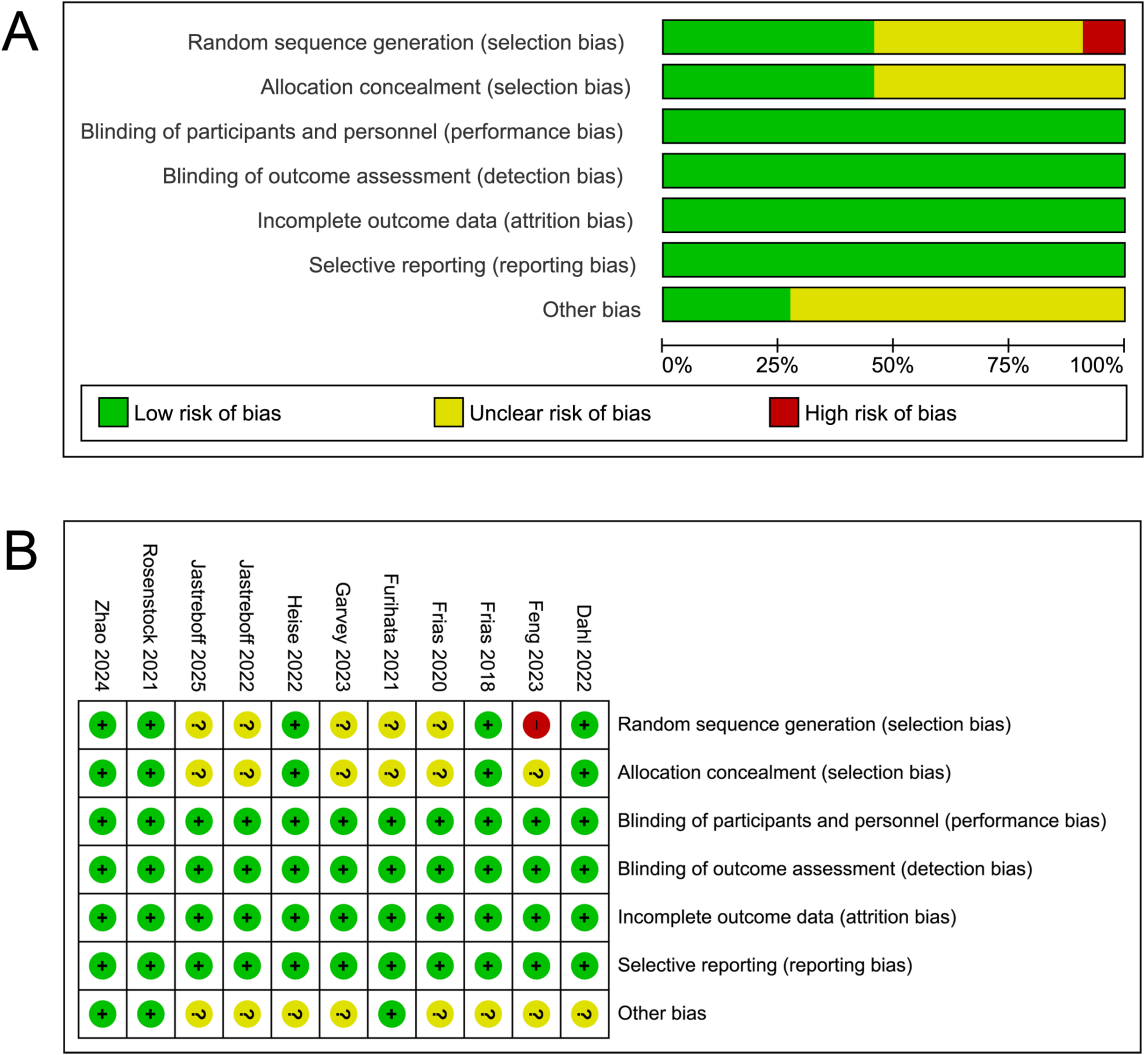
Risk assessment. **(A)** Risk of bias summary. **(B)** Graphs for risk of bias for studies.

Risk of bias assessment, as illustrated in **Figures 2A, B**, was conducted across multiple domains. The overall assessment revealed that while most studies exhibited a low risk of bias, certain methodological limitations were identified. Selection bias was generally well controlled, with a majority of studies implementing appropriate random sequence generation and allocation concealment. However, some studies did not report sufficient details on their randomization process leading to an unclear risk of bias. Most studies appropriately handled performance, detection, attrition and reporting bias, resulting in a low risk of attrition bias. Additionally, other potential sources of bias were identified in a subset of studies but were not consistently addressed.

### Meta-analysis of body weight change

**Figure 3:**
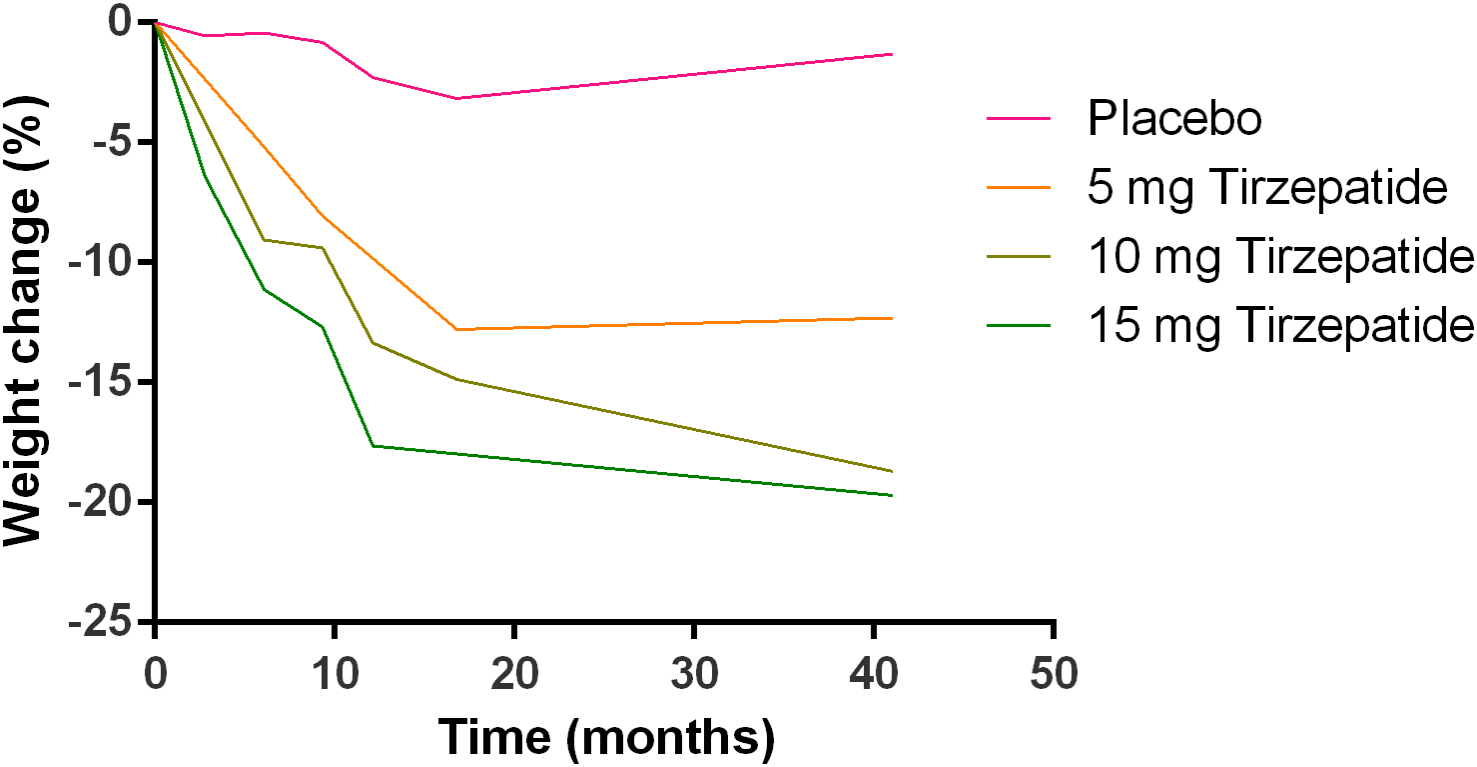
Profiles of body weight changes in different treatment groups are summarized from the included studies.

The changes in the body weight in different groups as summarized from all the studies included were shown in **Figure 3**. Tirzepatide caused the body weight loss in a dose-dependent manner.

**Figure 4:**
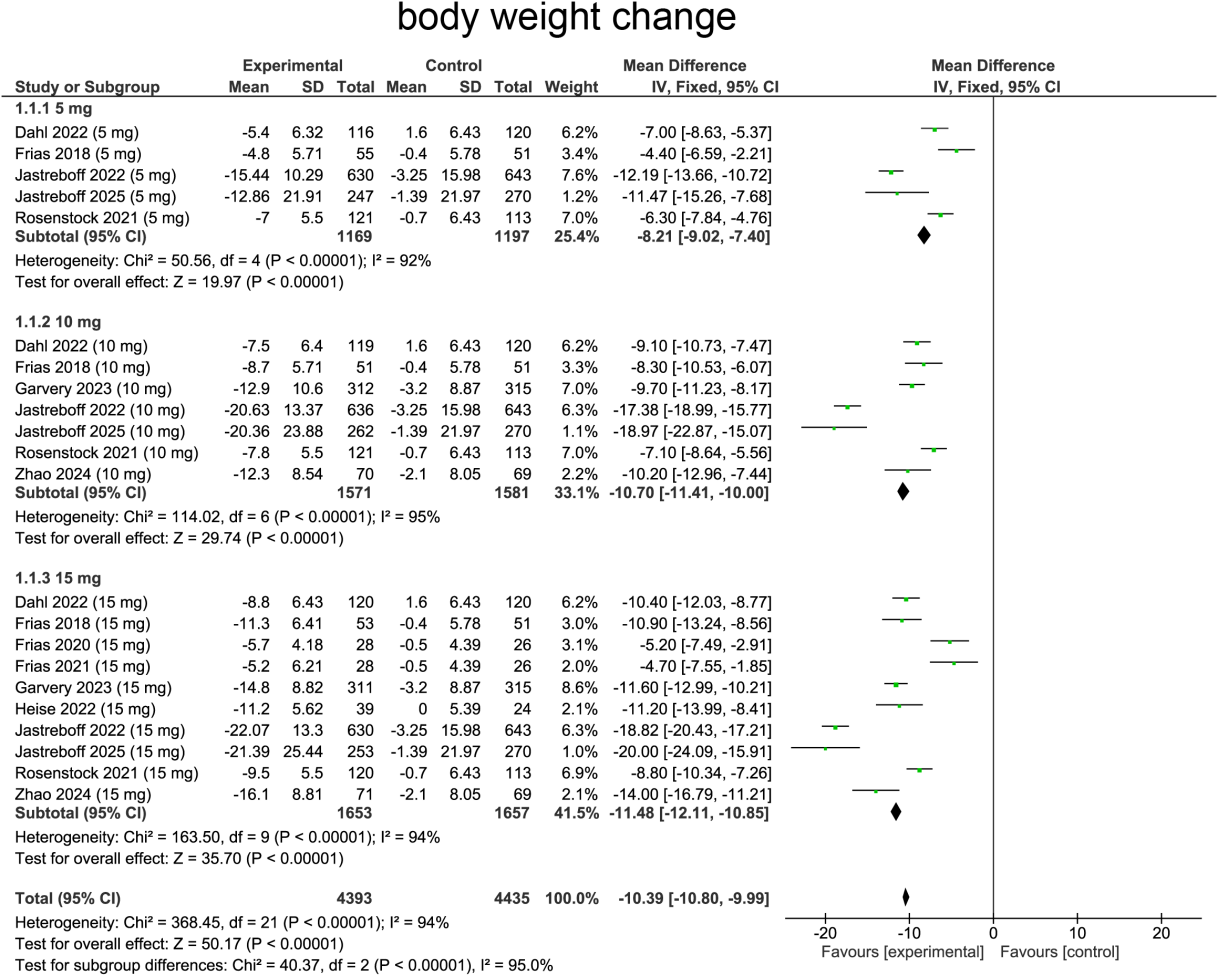
Meta-analysis of forest plot. Forest plot of body weight change.

Our meta-analysis evaluated effects of tirzepatide on body weight changes comparing with baseline. Heterogeneity was assessed using the I² statistic, which suggested heterogeneity (I²=94%, p<0.0001), providing rationale for employing random-effects model. Pooled analysis demonstrated a reduction in body weight in patients receiving tirzepatide comparing with placebo (WMD=-10.80, 95%CI:-10.80 to -9.99, p < 0.00001), indicating a substantial effect of the treatment (**Figure 4**).

For subgroup analysis, patients receiving tirzepatide 5 mg experienced body weight reduction comparing with placebo (WMD=-8.21, 95%CI:-9.02 to -7.40, p<0.0001, **Figure 4**). The 10 mg group exhibited a more pronounced effect (WMD=-10.70, 95% CI:-11.41 to -10.00, p<0.00001, **Figure 4**), indicating enhanced efficacy with increased dosage. The 15 mg group showed the greatest weight reduction, with a substantial and highly significant effect (WMD=-11.48, 95%CI:-12.11 to -10.85, p<0.0001, **Figure 4**), confirming a strong dose-response relationship.

Subgroup analyses by diabetes status mirrored this pattern: among patients with type 2 diabetes, mean weight changes were –6.17 kg (95% CI: –7.16 to –5.17, p < 0.00001) at 5 mg, –8.57 kg (95% CI: –9.41 to –7.74, p < 0.00001) at 10 mg, and –9.60 kg (95% CI: –10.32 to –8.89, p < 0.00001) at 15 mg. In non-diabetic participants, weight losses were larger: –12.10 kg (95% CI: –13.47 to –10.72, p < 0.00001) for 5 mg, –15.94 kg (95% CI: –17.25 to –14.62, p < 0.00001) for 10 mg, and –17.86 kg (95% CI: –19.19 to –16.54, p < 0.00001) for 15 mg, underscoring greater absolute weight reduction in non-diabetic cohorts (**Supplementary Table S1**).

### Meta-analysis of weight loss ≥ 5%, 10% and 15%

**Figure 5:**
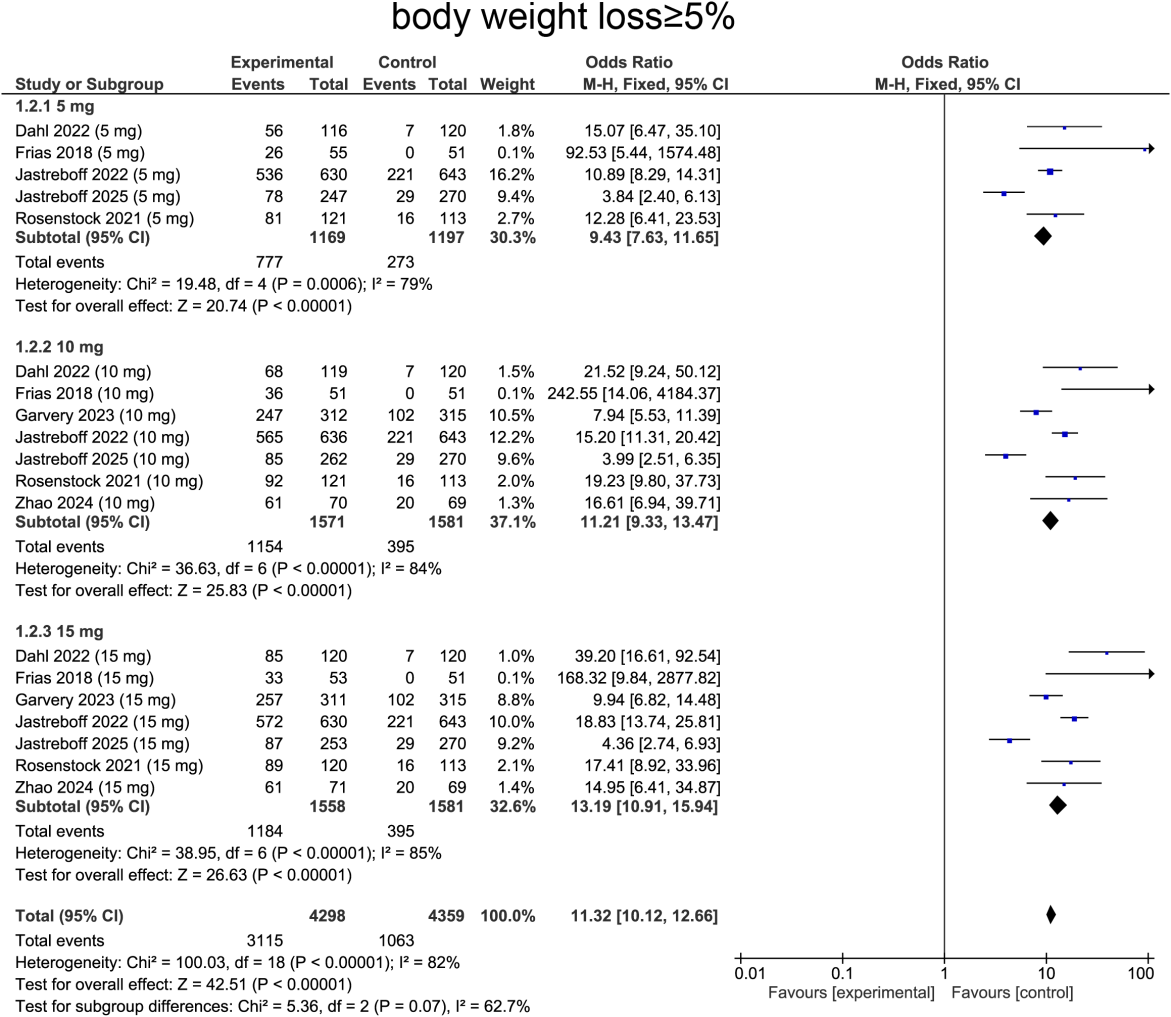
Meta-analysis of forest plot. Forest plot of body weight loss≥5%.

For weight loss ≥ 5%, heterogeneity was determined by I² statistic, which suggested heterogeneity (I²=82%, p<0.0001), providing rationale for employing random-effects model. Aggregated data indicated that tirzepatide treatment was correlated with higher likelihood of achieving ≥5% body weight loss comparing with placebo (OR=11.32, 95%CI:10.12 to 12.66, p<0.0001, **Figure 5**).

For subgroup analysis, patients receiving tirzepatide 5 mg showed increase in individuals achieving ≥5% weight loss comparing with placebo (OR=9.43, 95%CI:7.63 to 11.65, p<0.0001, **Figure 5**). The 10 mg group exhibited a more pronounced effect (OR=11.21, 95%CI:9.33 to 13.47, p<0.0001, **Figure 5**). The 15 mg group showed the greatest impact, with a substantially higher proportion of patients achieving ≥5% weight loss (OR=13.19, 95%CI:10.91 to 15.94, p<0.0001, **Figure 5**), confirming a strong dose-response relationship.

When stratified by diabetes status, type 2 diabetic patients exhibited ORs of 15.70 (95% CI: 9.49–25.98, p < 0.00001) at 5 mg, 12.20 (95% CI: 9.14–16.27, p < 0.00001) at 10 mg, and 15.01 (95% CI: 11.15–20.21, p < 0.00001). Among non‐diabetic participants, the corresponding ORs were 8.30 (95% CI: 6.56–10.49, p < 0.00001), 10.62 (95% CI: 8.37–13.46, p < 0.00001), and 12.13 (95% CI: 9.48–15.51, p < 0.00001) at 5, 10, and 15 mg, respectively (**Supplementary Table S1**).

**Figure 6:**
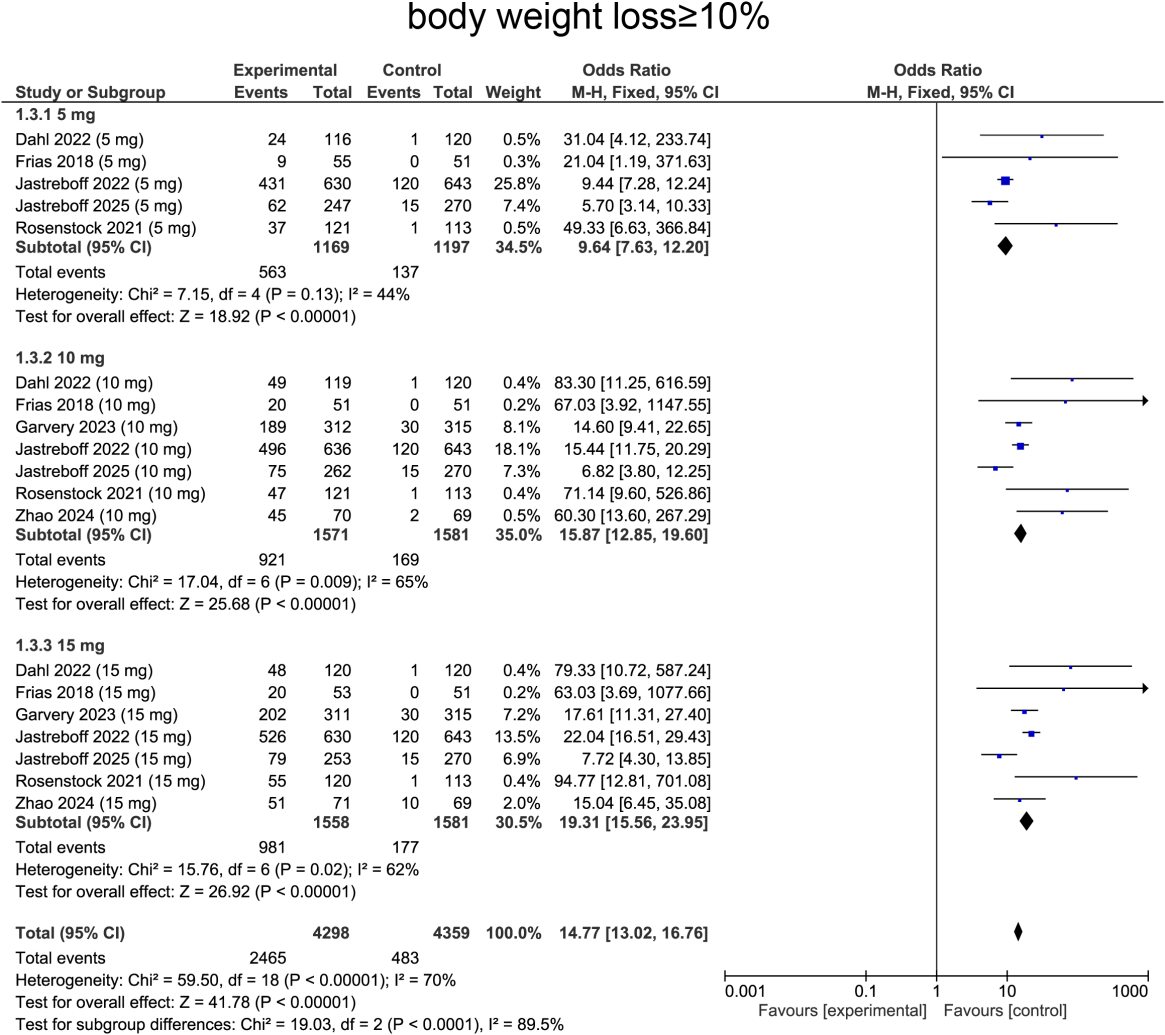
Meta-analysis of forest plot. Forest plot of body weight loss≥10%.

For weight loss ≥ 10%, heterogeneity was determined by I² statistic, which suggested heterogeneity (I² = 70%, p < 0.0001), providing rationale for employing random-effects model. Aggregated data indicated that tirzepatide treatment was correlated with a significantly higher likelihood of achieving ≥10% body weight loss comparing with placebo (OR=14.77, 95%CI:13.02 to 16.76, p<0.0001, **Figure 6**).

For subgroup analysis, patients receiving tirzepatide 5 mg showed increase in individuals achieving ≥10% weight loss comparing with placebo (OR=9.64, 95%CI: 7.63 to 12.20, p<0.0001, **Figure 6**). The 10 mg group exhibited a more pronounced effect (OR= 15.87, 95%CI:12.85 to 19.60, p<0.0001, **Figure 6**). The 15 mg group showed similar effects with 10 mg group, with a substantially higher proportion of patients achieving ≥10% weight loss (OR=19.31, 95%CI:15.56 to 23.95, p<0.0001, **Figure 6**).

For the ≥ 10% weight-loss threshold, the overall OR was 19.88 (95% CI: 14.53–27.21, p < 0.0001). In diabetic patients, ORs were 35.62 (95% CI: 10.00–126.86, p < 0.00001) at 5 mg, 21.51 (95% CI: 14.23–32.51, p < 0.00001) at 10 mg, and 25.53 (95% CI: 16.80–38.79, p < 0.00001) at 15 mg. Non-diabetic participants showed ORs of 8.61 (95% CI: 6.77–10.93, p < 0.00001), 13.88 (95% CI: 10.86–17.74, p < 0.00001), and 17.02 (95% CI: 13.24–21.88, p < 0.00001) at 5, 10, and 15 mg, respectively (**Supplementary Table S1**).

**Figure 7:**
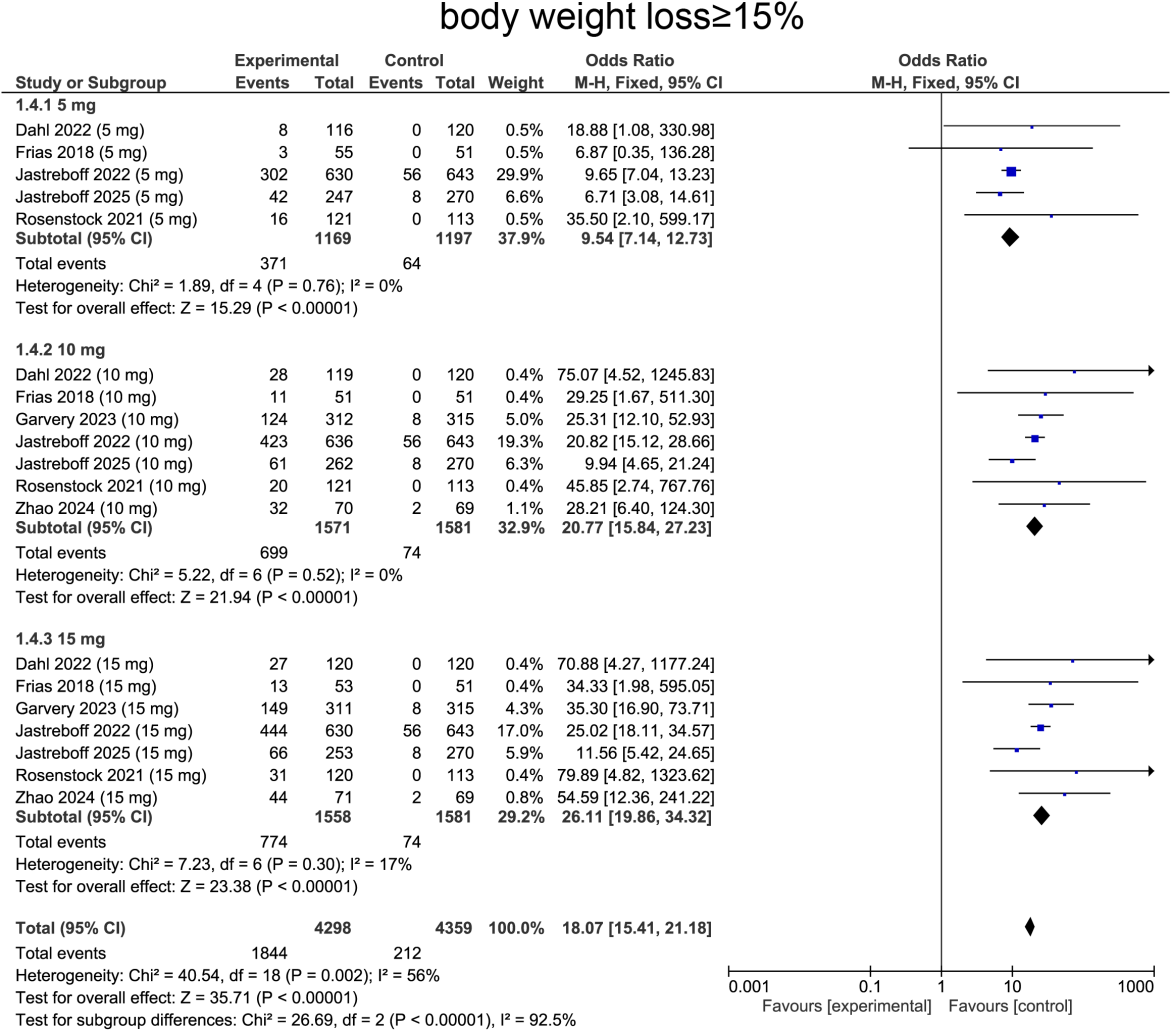
Meta-analysis of forest plot. Forest plot of body weight loss≥15%.

For weight loss ≥ 15%, heterogeneity was determined by I² statistic, which suggested heterogeneity (I² = 56%, p = 0.002), providing rationale for employing random-effects model. Aggregated data revealed that tirzepatide treatment was correlated with a significantly higher likelihood of achieving ≥15% body weight loss comparing with placebo (OR=18.07, 95%CI:15.41 to 21.18, p<0.0001, **Figure 7**).

For subgroup analysis, patients receiving tirzepatide 5 mg showed increase in individuals achieving ≥15% weight loss comparing with placebo (OR=9.54, 95%CI: 7.14 to 12.73, p<0.0001, **Figure 7**). The 10 mg group exhibited a more pronounced effect (OR=20.77, 95%CI:15.84 to 27.23, p<0.0001, **Figure 7**). The 15 mg group showed the greatest impact, with a substantially higher proportion of patients achieving ≥15% weight loss (OR=26.11, 95%CI:19.86 to 34.32, p<0.0001, **Figure 7**), confirming a strong dose-response relationship.

Finally, for achieving ≥ 15% weight loss, the pooled OR was 23.13 (95% CI: 16.17–33.07, p < 0.0001). Diabetic patients demonstrated ORs of 20.03 (95% CI: 3.87–103.64, p < 0.00001) at 5 mg, 30.19 (95% CI: 15.33–59.44, p < 0.00001) at 10 mg, and 41.04 (95% CI: 20.76–81.11, p < 0.00001) at 15 mg. In the non-diabetic subgroup, corresponding ORs were 9.12 (95% CI: 6.80–12.23, p < 0.00001), 18.58 (95% CI: 13.86–24.90, p < 0.00001), and 22.65 (95% CI: 16.86–30.44, p < 0.00001) at 5, 10, and 15 mg, respectively (**Supplementary Table S1**).

### Incidence of adverse events

**Figure 8:**
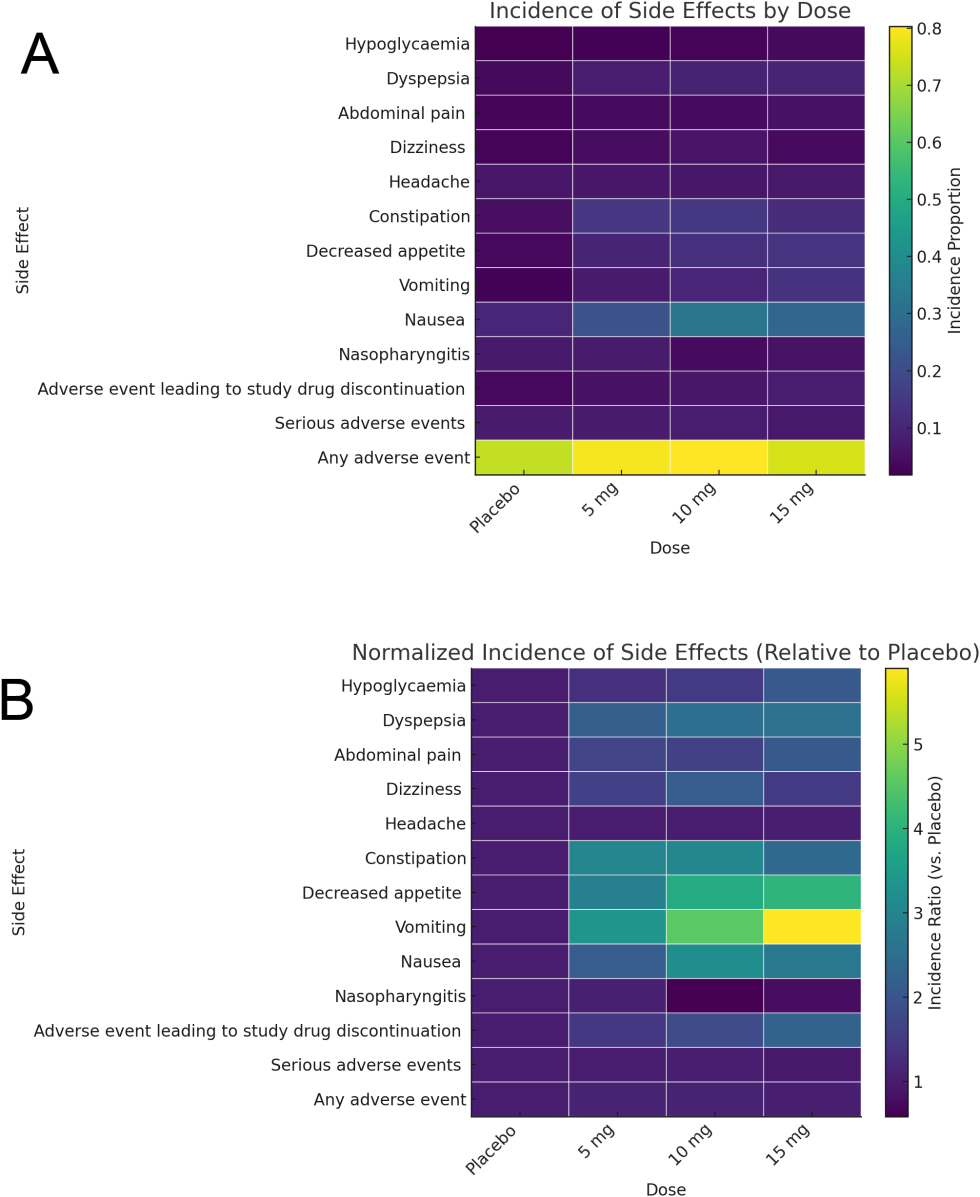
Profiles of adverse events as illustrated by heatmap. **(A)** Incidence of adverse events in different groups. **(B)** Normalized adverse events incidence in different groups.

The incidence heatmap shows that gastrointestinal side effects, particularly nausea, vomiting, and dyspepsia, rise markedly with tirzepatide dose (e.g., nausea increases from ~10% on placebo to ~30% at 15 mg), while non-GI events remain low (<10%) across all arms. Serious adverse events and drug discontinuations stay below 5% for all doses, highlighting an overall favorable safety profile (**Figure 8A**). When normalized to placebo, decreased appetite exhibits the greatest fold-increase (~3× at 15 mg), followed by vomiting (~2.5×) and nausea (~2×), whereas non-GI effects show minimal relative change (<1.5×) (**Figure 8B**). Together, these heatmaps underscore that tirzepatide’s dose-dependent tolerability concerns are driven primarily by gastrointestinal adverse events.

### Meta-analysis of any adverse event

For any adverse event, heterogeneity was determined by I² statistic, which suggested heterogeneity (I²=62%, p<0.0001), providing rationale for employing random-effects model. Aggregated data indicated that patients receiving tirzepatide had a higher likelihood of experiencing adverse effects comparing with placebo group (OR=1.34, 95%CI:1.21 to 1.47, p<0.0001, **Supplementary Figure S1**). For subgroup analysis, patients receiving tirzepatide 5 mg reported higher incidence of adverse events comparing with placebo (OR=1.48, 95%CI:1.22 to 1.80, p<0.0001, **Supplementary Figure S1**). Similar results were found in 10 and 15 mg subgroups.

Subgroup analysis by diabetic status (**Supplementary Table S2**) revealed that among diabetic patients, the 5 mg dose showed a non-significant trend toward more adverse events (OR=1.38, 95% CI 0.97–1.96, p = 0.05), whereas non-diabetic patients at 5 mg experienced a significant increase (OR=1.53, 95% CI 1.22–1.92, p = 0.0003). At 10 mg, diabetic patients had a modest yet significant increase (OR=1.19, 95% CI 0.92–1.53, p = 0.0003), while non-diabetics showed an even larger effect (OR=1.75, 95% CI 1.40–2.20, p < 0.00001). At 15 mg, both diabetic (OR 1.26, 95% CI 1.00–1.59, p < 0.00001) and non-diabetic (OR=1.05, 95% CI 0.85–1.30, p = 0.63) subgroups trended in the same direction, though statistical significance was confined to the diabetic cohort.

### Meta-analysis of serious adverse events and advent events leading to study drug discontinuation

For SAEs, heterogeneity was determined by I² statistic, which suggested no heterogeneity (I²=0%, p=0.93), providing rationale for employing random-effects model. Aggregated data revealed no potentiation in risk of SAEs with tirzepatide treatment comparing with placebo (OR=0.97, 95%CI:0.83-1.14, p=0.74, **Supplementary Figure S2A**), indicating that the treatment was generally well tolerated. Subgroup analysis by tirzepatide dose demonstrated that SAEs did not differ among dosing groups (**Supplementary Figure S2A**). Diabetic and non-diabetic subgroups likewise showed no significant differences in SAE risk at any dose (**Supplementary Table S2**).

For adverse events leading to treatment discontinuation, heterogeneity was determined by I² statistic, which suggested no heterogeneity (I²= 8%, p=0.18), providing rationale for employing fixed-effects model. Aggregated data indicated that tirzepatide group had higher likelihood of discontinuing treatment comparing with placebo (OR=1.98, 95%CI: 1.63 to 2.61, p<0.0001, **Supplementary Figure S2B**).

For subgroup analysis, a dose-response relationship was observed, with higher tirzepatide doses associated with greater treatment discontinuation rates. The 5 mg group exhibited a lower discontinuation rate (OR=1.57, 95%CI:1.05 to 2.36, p=0.03, **Supplementary Figure 2B**), while the 10 mg and 15 mg groups demonstrated a significantly higher likelihood of treatment withdrawal due to adverse events (10 mg: OR=1.93, 95%CI: 1.38 to 2.69, p=0.0001; 15 mg: OR=2.31, 95%CI:1.69 to 3.14, p<0.0001, **Supplementary Figure S2B**). In diabetic patients, only the 15 mg dose significantly increased discontinuations (OR=2.17, 95% CI 1.37–3.45, p = 0.001), whereas in non-diabetics both 10 mg (OR=2.26, 95% CI 1.48–3.43, p = 0.0001) and 15 mg (OR=2.42, 95% CI 1.59–3.67, p < 0.0001) were significant (**Supplementary Table S2**).

### Meta-analysis of nausea and vomiting

For nausea, heterogeneity was determined by I² statistic, which suggested no heterogeneity (I²=70%, p<0.00001), providing rationale for employing random-effects model. Aggregated data indicated that patients treated with tirzepatide had a higher incidence of nausea comparing with placebo (OR=3.08, 95%CI:3.74 to 3.46, p<0.0001, **Supplementary Figure S3A**). For subgroup analysis, 5 mg group exhibited a higher incidence of nausea comparing with placebo (OR=2.24, 95%CI:1.78 to 2.81, p<0.0001, **Supplementary Figure S3A**), while 10 and 15 mg groups demonstrated a significantly higher incidence of nausea (10 mg: OR=3.45, 95 CI:2.82 to 4.21, p<0.0001; 15 mg: OR=3.45, 95%CI:2.84 to 4.18, p<0.0001, **Supplementary Figure S3A**). In diabetics, ORs ranged from 3.28 (5 mg) to 4.32 (15 mg); in non-diabetics, from 2.10 (5 mg) to 3.32 (10 mg) (**Supplementary Table S2**).

For vomiting, heterogeneity was determined by I² statistic, which suggested no heterogeneity (I²=0%, p=0.94), providing rationale for employing fixed-effects model. Aggregated data indicated that patients treated with tirzepatide had a higher incidence of nausea comparing with placebo (OR=5.51, 95%CI:4.40 to 6.91, p<0.0001, **Supplementary Figure S3B**). For subgroup analysis, patients receiving tirzepatide 5 mg had an increase in vomiting events comparing with placebo (OR=5.06, 95%CI:3.48 to 7.36, p<0.0001, **Supplementary Figure S3B**). The 10 mg group exhibited a more pronounced effect (OR=6.56, 95%CI:461 to 9.34, p<0.0001, **Supplementary Figure S3B**). The 15 mg group showed the greatest impact, with a substantially higher proportion of patients experiencing vomiting (OR=6.06, 95%CI:4.16 to 8.82, p<0.0001, **Supplementary Figure S3B**), confirming a strong dose-response relationship. In diabetic patients, the effect grew from OR 2.73 at 5 mg to OR 5.08 at 15 mg; non-diabetics showed an even steeper dose-response from OR 5.27 to OR 8.24 (**Supplementary Table S2**).

### Meta-analysis of constipation and abdominal pain

For constipation, heterogeneity was determined by I² statistic, which suggested no heterogeneity (I²=0%, p=0.78), providing rationale for employing fixed-effects model. Aggregated data indicated that patients treated with tirzepatide had a higher incidence of constipation comparing with placebo (OR=3.10, 95%CI:2.55 to 3.75, p<0.0001, **Supplementary Figure S4A**).

For subgroup analysis, patients receiving tirzepatide 5 mg reported a greater incidence of constipation comparing with placebo group (OR=3.08, 95%CI:2.28 to 4.17, p<0.0001, **Supplementary Figure S4A**). Similar results were observed in10 mg and 15 mg subgroups (**Supplementary Figure S4A**). In diabetics, the 5 mg effect was particularly pronounced (OR 3.39), while non-diabetics had ORs from 3.05–3.30 (**Supplementary Table S2**).

For abdominal pain, heterogeneity was determined by I² statistic, which suggested no heterogeneity (I²=0%, p=0.97), providing rationale for employing fixed-effects model. Aggregated data indicated that patients treated with tirzepatide had a higher incidence of abdominal pain comparing with placebo (OR=1.85, 95%CI:1.47 to 2.34, p<0.0001; **Supplementary Figure S4B**). For subgroup analysis, 5 mg group showed potential risk (OR=1.72, 95%CI:1.09 to 2.71, p=0.02, **Supplementary Figure S4B**), while 10 and 15 mg groups exhibited higher likelihood of experiencing abdominal pain (10 mg: OR=1.76, 95%CI:1.18 to 2.63, p=0.006; 15 mg: OR=2.02, 95%CI: 1.40 to 2.92, p=0.0002, **Supplementary Figure S4B**). Among diabetics only the 15 mg dose was significant (OR=2.97, p = 0.0005), while non-diabetics showed significant effects at 5 mg (OR=1.75) and 10 mg (OR=1.77) (**Supplementary Table S2**).

### Meta-analysis of decreased appetite and dyspepsia

For decreased appetite, heterogeneity was determined by I² statistic, which suggested no heterogeneity (I²=0%, p=0.78), providing rationale for employing fixed-effects model. Aggregated data indicated that patients treated with tirzepatide had a higher incidence of decreased appetite comparing with placebo (OR=4.28, 95%CI:3.53 to 5.18, p<0.0001, **Supplementary Figure S5A**). For subgroup analysis, patients receiving tirzepatide 5 mg reported a greater incidence of decreased appetite comparing with placebo group (OR=3.67, 95%CI: 2.49 to 5.42, p<0.0001, **Supplementary Figure S5A**). Similar results were found in the 10 mg and 15 mg subgroups. When stratified by diabetic status, diabetic participants experienced ORs ranging from 7.10 to 7.55 across the three doses, whereas non-diabetic participants showed ORs of 2.98 to 3.81 (all p < 0.001) (**Supplementary Table S2**).

For dyspepsia, heterogeneity was determined by I² statistic, which suggested no heterogeneity (I²=0%, p=0.97), providing rationale for employing fixed-effects model. Aggregated data indicated that patients treated with tirzepatide had a higher incidence of dyspepsia comparing with placebo (OR=2.50, 95 CI:2.07 to 3.01, p<0.0001, **Supplementary Figure S5B**). For subgroup analysis, patients receiving tirzepatide 5 mg reported a greater incidence of dyspepsia comparing with placebo group (OR=2.12, 95%CI:1.49 to 3.03, p<0.0001, **Supplementary Figure S5B**). Similar results were observed in 10 and 15 mg subgroups. In diabetic patients, ORs ranged from 2.41 to 3.26, while non-diabetic patients had ORs between 1.94 and 2.87 (all p < 0.01) (**Supplementary Table S2**).

### Meta-analysis of dizziness, headache, hypoglycemia and nasopharyngitis

For dizziness, heterogeneity was determined by I² statistic, which suggested no heterogeneity (I²=0%, p=0.83), providing rationale for employing fixed-effects model. Aggregated data indicated that patients treated with tirzepatide had a higher incidence of dizziness comparing with placebo (OR=1.81, 95%CI:1.40 to 2.35, p<0.0001, **Supplementary Figure S6A**). For the subgroup analysis, the 5 mg group showed no potential risk (OR=1.56, 95%CI:0.95 to 2.57, p=0.06, **Supplementary Figure S6A**), while the 10 mg and 15 mg groups exhibited a significantly higher likelihood of experiencing abdominal pain (10 mg: OR=2.40, 95%CI:1.57 to 3.67, p<0.001; 15 mg: OR=1.49, 95%CI:0.97 to 2.30, p=0.04, **Supplementary Figure S6A**). The 5 mg dose was not significantly associated with dizziness in either diabetic (OR=0.92, 95% CI: 0.13–6.82, p>0.05) or non-diabetic populations (OR=1.62, 95% CI: 0.97–2.70, p>0.05). However, a significantly increased risk was observed for the 10 mg dose, particularly in the non-diabetic group (OR=2.28, 95% CI: 1.40–3.70, p=0.0008). The 15 mg dose showed a trend toward increased risk in both subpopulations but did not reach statistical significance (**Supplementary Table S2**).

For headache, heterogeneity was determined by I² statistic, which suggested no heterogeneity (I²=6%, p=0.52), providing rationale for employing fixed-effects model. Aggregated indicated that patients treated with tirzepatide had no risk of headache comparing with placebo (OR=1.00, 95%CI:0.84 to 1.20, p=0.97, **Supplementary Figure S6B**). The subgroup analysis showed similar findings. Subgroup analysis confirmed consistent findings across all dose groups and both diabetic and non-diabetic patients (**Supplementary Table S2**).

For hypoglycemia, heterogeneity was determined by I² statistic, which suggested heterogeneity (I²=65%, p<0.0001), providing rationale for employing random-effects model. Aggregated data indicated that patients treated with tirzepatide had no risk of hypoglycemia comparing with placebo (OR= 2.05, 95%CI:0.95 to 4.42, p=0.07, **Supplementary Figure S6C**). The subgroup analysis showed similar findings. Among non-diabetic patients, a significant increase in hypoglycemia was observed with the 5 mg dose (OR=9.30, 95% CI: 1.18–73.66, p=0.03). In diabetic patients, none of the dose groups demonstrated a significant effect (**Supplementary Table S2**).

For nasopharyngitis, heterogeneity was determined by I² statistic, which suggested heterogeneity (I²=0%, p=0.71), providing rationale for employing fixed-effects model. Aggregated data indicated that patients treated with tirzepatide had lower risk of nasopharyngitis comparing with placebo (OR=0.71, 95%CI:0.50 to 0.90, p=0.005, **Supplementary Figure S6D**). For the subgroup analysis,5 and 15 mg groups showed no potential risk (5 mg: OR=0.93, 95%CI:0.59 to 1.45, p=0.74; 15 mg: OR=0.70, 95%CI: 0.47 to 1.03, p=0.07, **Supplementary Figure S6D**), while the 10 mg group exhibited a significantly lower likelihood of experiencing nasopharyngitis (OR=0.58, 95%CI:0.38 to 0.88, p=0.01, **Supplementary Figure 1S6D**). A significant reduction in the 10 mg dose group (OR=0.49, 95% CI: 0.30–0.79, p=0.004), particularly in diabetic patients. However, the 5 mg (OR=0.77, 95% CI: 0.45–1.32, p=0.35) and 15 mg groups (OR=0.73, 95% CI: 0.48–1.11, p=0.14) did not demonstrate statistically significant differences from placebo (**Supplementary Table S2**).

### Publication bias

Funnel plots of our meta-analysis are shown in **Supplementary Figures S7**-**10**, and funnel plot results indicated no publication bias.

## Discussion

This meta-analysis provides a comprehensive evaluation of tirzepatide’s efficacy and safety for body weight reduction, incorporating data from ten clinical trials conducted across diverse populations. The pooled analysis demonstrated that tirzepatide treatment led to a significant reduction in body weight comparing with placebo, with a dose-dependent response, where higher doses resulted in greater weight loss effects. Additionally, the drug significantly increased the proportion of patients achieving ≥5%, ≥10%, and ≥15% weight loss, reinforcing its potential as a pharmacological intervention for obesity and type 2 diabetes management. These findings are particularly relevant given the growing need for effective weight loss treatments that can complement lifestyle interventions and provide sustained metabolic benefits. However, while tirzepatide demonstrated superior efficacy comparing with placebo, an increased risk of adverse events, particularly gastrointestinal symptoms, was observed. These side effects were dose-dependent, with higher doses being associated with greater treatment discontinuation rates. Nonetheless, serious adverse events were not significantly different from placebo, suggesting that tirzepatide remains a well-tolerated therapy overall.

Our findings align with previous studies evaluating GLP-1 receptor agonists for weight management, including semaglutide and liraglutide (31–34). The mean weight loss achieved with tirzepatide in our meta-analysis (ranging from 7.5 kg with 5 mg to 10.7 kg with 15 mg) is comparable to or exceeds the weight loss reported with semaglutide (approximately 6.0–12.5 kg) in prior trials (34, 35). The greater efficacy of tirzepatide may be attributed to its dual mechanism of action, as a GLP-1 and GIP receptor agonist, which enhances insulin sensitivity, promotes satiety, and reduces energy intake (16, 36, 37). This dual incretin effect is thought to provide superior metabolic benefits comparing with single GLP-1 receptor agonists like semaglutide (13, 38), potentially explaining the enhanced weight loss effects observed in this analysis. Our findings are consistent with prior clinical trials, which reported substantial weight loss with tirzepatide across multiple dosing regimens, further supporting its superior efficacy comparing with single-receptor GLP-1 agonists (21, 22).

The dose-dependent effect observed in this meta-analysis further confirms previous reports on tirzepatide’s efficacy (36, 37, 39). Patients receiving higher doses (15 mg) achieved the greatest weight loss, while the 10 mg and 5 mg groups exhibited progressively smaller effects. This trend aligns with findings from previous studies, which demonstrated that higher tirzepatide doses elicit greater metabolic and weight reduction benefits, likely due to increased activation of GLP-1 and GIP receptors, leading to greater appetite suppression and energy expenditure (36, 37, 39). Furthermore, our analysis of ≥5%, ≥10%, and ≥15% weight loss proportions demonstrated a clear dose-response relationship, reinforcing tirzepatide’s strong efficacy profile for weight management. These findings suggest that tirzepatide may offer a greater therapeutic advantage over other weight loss pharmacotherapies, particularly for patients who require more substantial weight reduction.

Despite its efficacy, tirzepatide was associated with a higher incidence of adverse effects comparing with placebo, particularly gastrointestinal symptoms such as nausea, vomiting, and constipation. These findings are consistent with reports on other GLP-1 receptor agonists, including semaglutide and liraglutide, which also demonstrate a high prevalence of nausea and vomiting as common treatment-related side effects (40, 41). The underlying mechanism for these effects is likely related to slowed gastric emptying and central appetite regulation mediated by GLP-1 activation (42, 43). Importantly, while tirzepatide increased treatment discontinuation due to adverse effects, our analysis confirmed that serious adverse events were not significantly different from placebo, indicating a favorable overall safety profile. This suggests that while gastrointestinal side effects may impact adherence, they are unlikely to pose significant safety concerns. Moreover, prior research suggests that gradual dose titration and supportive care strategies may help mitigate gastrointestinal symptoms, improving treatment tolerability.

The presence of heterogeneity in the meta-analysis of body weight change suggests variability among studies, likely due to differences in population characteristics, trial duration, and treatment regimens. Previous meta-analyses on GLP-1 receptor agonists for obesity treatment have similarly reported high heterogeneity (44), indicating that weight loss responses to these agents are influenced by multiple factors, including baseline BMI, metabolic status, and adherence to treatment. For example, studies involving patients with type 2 diabetes often report smaller weight reductions comparing with non-diabetic individuals, likely due to insulin resistance and differences in metabolic regulation (45). Our findings underscore the need for further investigation into patient subgroups who may benefit most from tirzepatide, particularly in real-world clinical settings, where adherence, lifestyle modifications, and comorbidities may influence outcomes.

While our systematic review and meta-analysis offer a comprehensive synthesis of tirzepatide’s efficacy and safety based on recent clinical trials, several limitations should be acknowledged that may affect the interpretation and generalizability of the findings. First, heterogeneity across included studies was notable, particularly in outcomes such as body weight change. This variability likely reflects differences in baseline participant characteristics, including diabetes status, BMI, sex distribution, and background medications. Although random-effects models were used and subgroup analyses were conducted where feasible, such heterogeneity introduces uncertainty in the pooled estimates. Future meta-analyses stratified by more granular clinical factors may help refine these findings. Second, the inclusion of both patients with type 2 diabetes mellitus (T2D) and non-diabetic individuals introduces clinical complexity. Tirzepatide exerts distinct physiological effects in these populations, particularly in glycemic control, which may influence both efficacy and safety outcomes. To address this, we conducted subgroup analyses stratified by diabetes status and reported differential responses in HbA1c reduction and weight loss. Nevertheless, the inherent differences in treatment goals and pathophysiology between these populations should be considered when interpreting our pooled results.

Third, the follow-up duration in most included trials was limited to 72 weeks or less. As such, our findings reflect short- to intermediate-term efficacy and tolerability. Long-term safety outcomes—including rare adverse events, cardiovascular benefits or risks, and sustained weight maintenance—remain uncertain. Although recent extension studies such as SURMOUNT-1 (172 weeks) provide valuable insights, many were published after our data cutoff and were only incorporated into the qualitative synthesis. Additional long-term and real-world studies are warranted to confirm the durability of tirzepatide’s effects. Fourth, all included trials were placebo-controlled. While this allows for standardized comparisons, it limits direct inference regarding tirzepatide’s performance relative to other agents such as semaglutide, which is widely regarded as a benchmark GLP-1 receptor agonist. Although a narrative contextual comparison with semaglutide was included, formal head-to-head trials or network meta-analyses are necessary to establish relative efficacy, tolerability, and cost-effectiveness. Fifth, real-world data on treatment adherence, patient-reported outcomes, and economic impact were not captured in the included RCTs. These dimensions are crucial for assessing the broader clinical and public health utility of tirzepatide. Further post-marketing surveillance and observational research will be important in evaluating its uptake, adherence patterns, and patient satisfaction in routine care settings. Sixth, some included studies did not clearly describe randomization procedures or allocation concealment, leading to an “unclear” risk of bias rating. While sensitivity analyses excluding such studies did not materially alter the results, transparency in reporting trial methodology remains a key requirement for future research. Finally, gastrointestinal adverse events—such as nausea, vomiting, and diarrhea—were common, particularly at higher tirzepatide doses (10–15 mg), and contributed to treatment discontinuation in some trials. While generally consistent with the known class effects of GLP-1 receptor agonists, these side effects may impact long-term adherence and should be carefully considered in clinical decision-making.

## Conclusions

Our meta-analysis confirms that tirzepatide is a highly effective pharmacologic intervention for weight reduction, with a strong dose-response relationship and a safety profile comparable to existing GLP-1 receptor agonists. The drug significantly increases the likelihood of achieving clinically meaningful weight loss thresholds, positioning it as a promising treatment for obesity and metabolic disorders. While gastrointestinal side effects remain a concern, their impact on adherence may be mitigated through dose titration strategies. Future research should focus on long-term outcomes, optimal patient selection, and real-world effectiveness to further establish tirzepatide’s role in obesity and diabetes management.

## Data Availability

The original contributions presented in the study are included in the article/**Supplementary Material**. Further inquiries can be directed to the corresponding authors.
