# Correction: Efficacy and safety of tirzepatide for weight loss in patients with obesity or type 2 diabetes: a systematic review and meta-analysis

**DOI:** 10.3389/fendo.2025.1683829

**Published:** 2025-08-25

**Authors:** Qiru Tian, Yi Song, Yan Deng, Shike Lin

**Affiliations:** ^1^ Hainan Vocational University of Science and Technology, Haikou, China; ^2^ Faculty of Medicine, Chinese University of Hong Kong, Hong Kong, Hong Kong SAR, China; ^3^ Office of Science and Technology, Youjiang Medical University for Nationalities, Baise, Guangxi, China; ^4^ Faculty of Nursing, Youjiang Medical University for Nationalities, Baise, Guangxi, China; ^5^ Department of Obstetrics and Gynaecology, Affiliated Hospital of Youjiang Medical University for Nationalities, Baise, Guangxi, China

**Keywords:** tirzepatide, weight loss, meta-analysis, obesity, type 2 diabetes, adverse events

Affiliation “^1^Hainan Vocational University of Science and Technology, Haikou, China” was erroneously given as “^1^ School of Basic Medical Sciences, Hainan Vocational University of Science and Technology, Haikou, China”.

The original version of this article has been updated.

