# Supplementary material for: Efficacy and safety of tirzepatide for weight loss in patients with obesity or type 2 diabetes: a systematic review and meta-analysis
Source: Front Endocrinol (Lausanne). 2025 Jul 17;16:1593134. doi: 10.3389/fendo.2025.1593134 (PMC12310450; doi:10.3389/fendo.2025.1593134)
Supplement: Supplementary file 1 [file DataSheet1.docx]

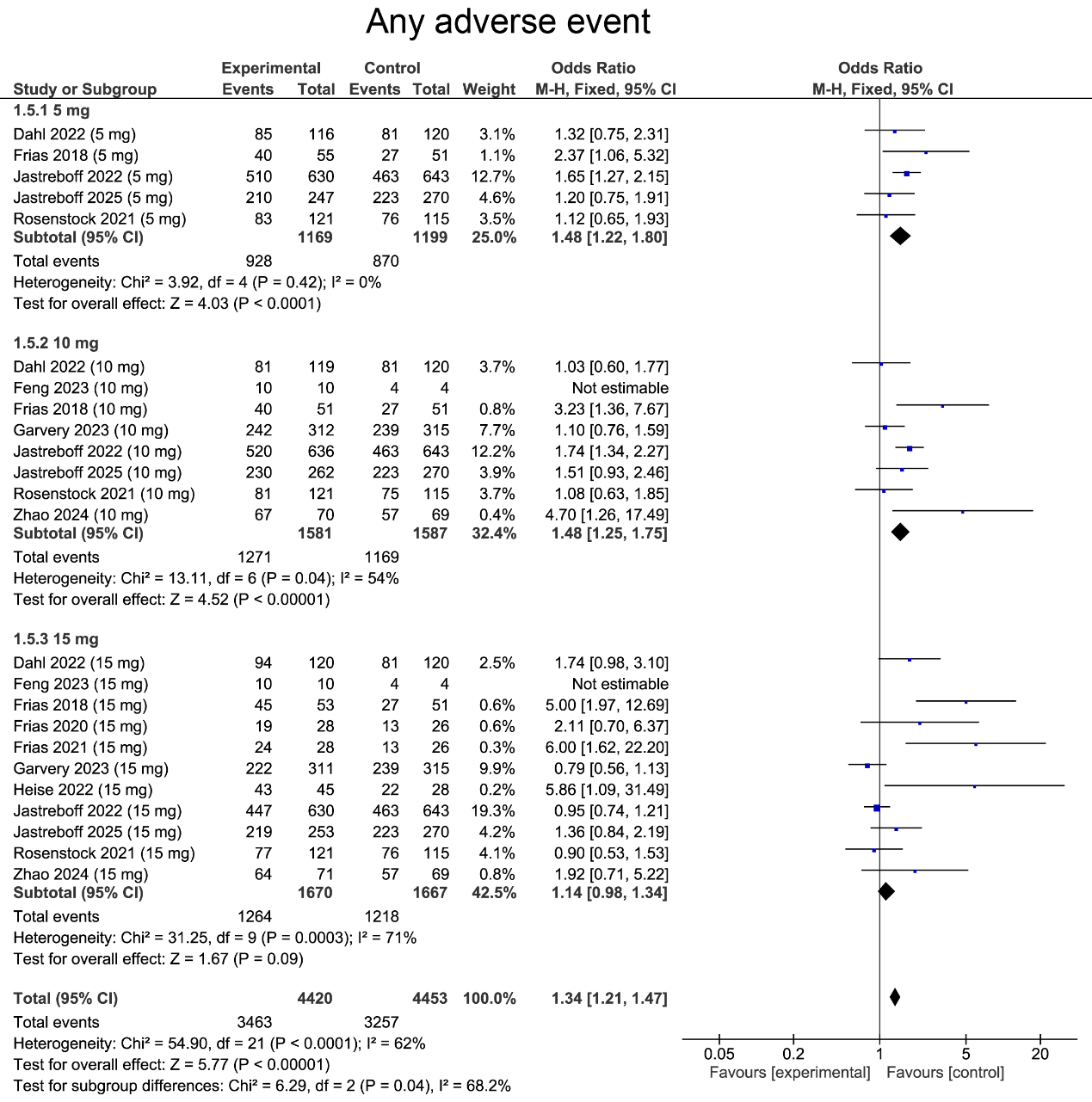


Supplemental Figure S1. Meta-analysis of forest plot. Forest plot of any adverse event.


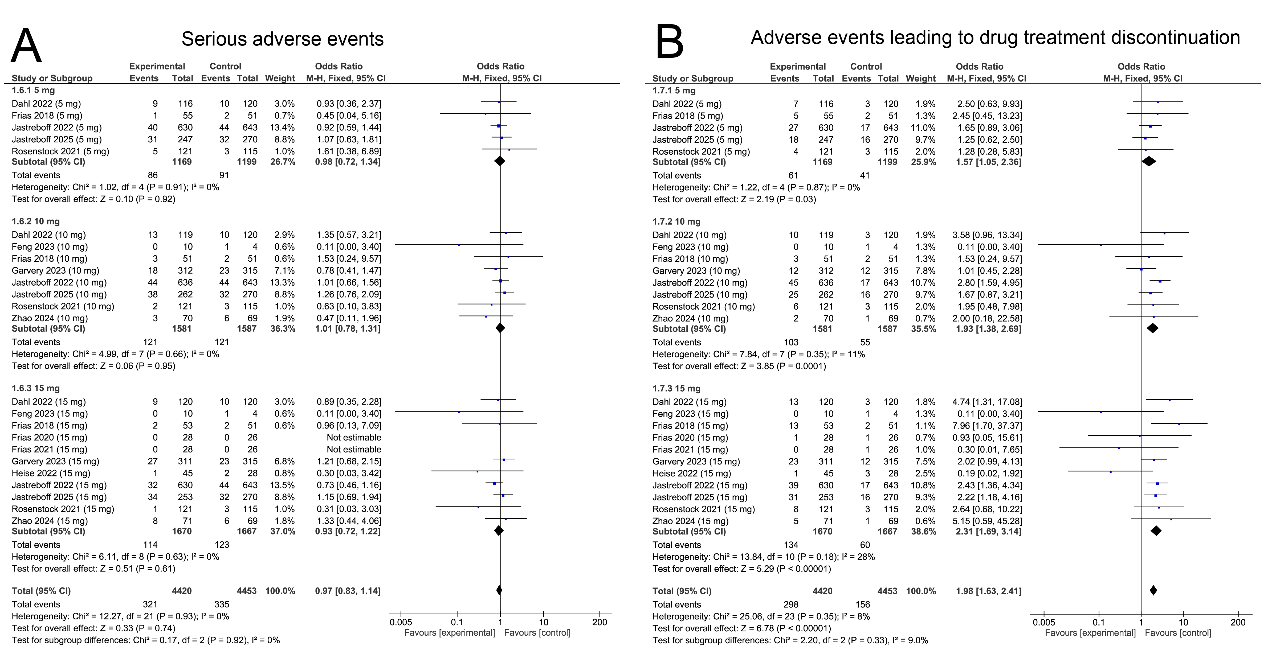


Supplemental Figure S2. Forest plot of serious adverse events (A) and adverse events leading to drug treatment discontinuation (B).


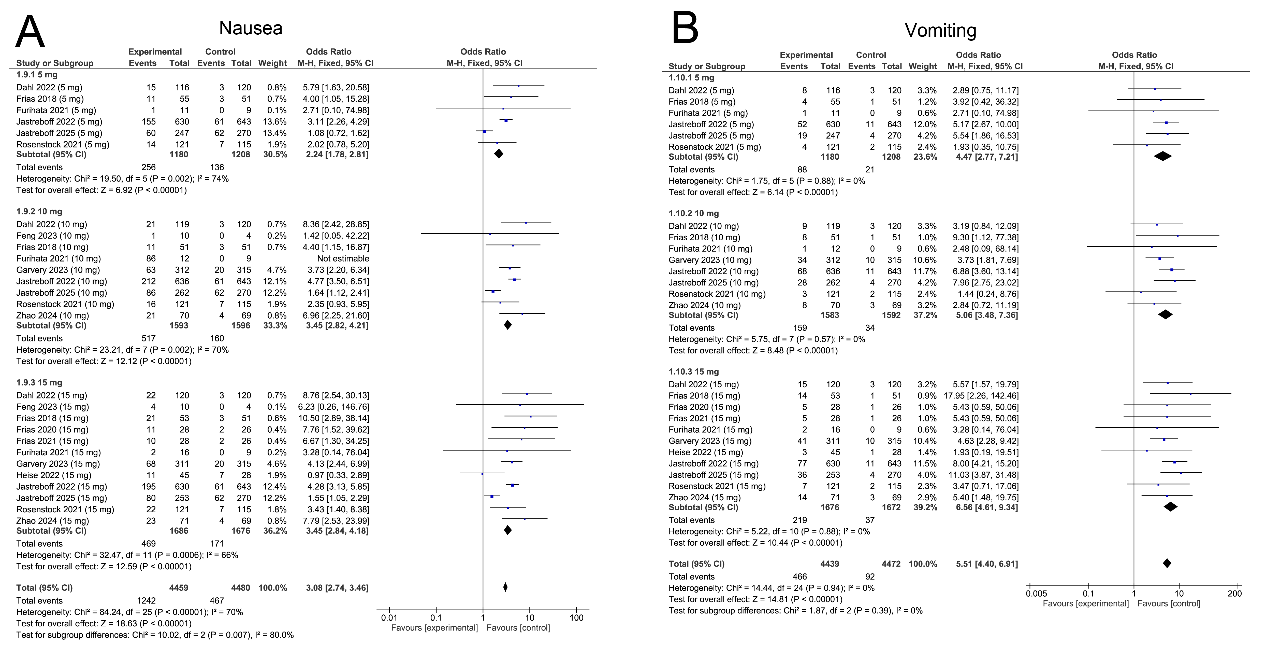


Supplemental Figure S3. Forest plot of nausea (A) and vomiting (B).


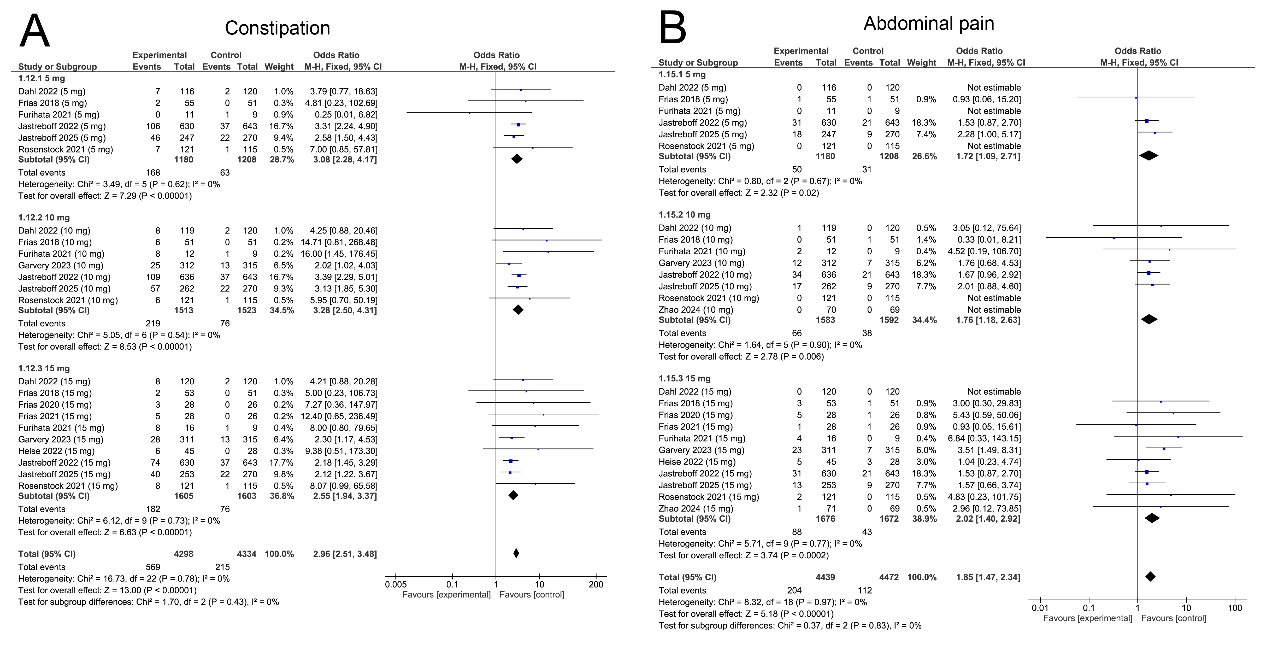


Supplemental Figure S4. Forest plot of constipation (A) and abdominal pain (B).


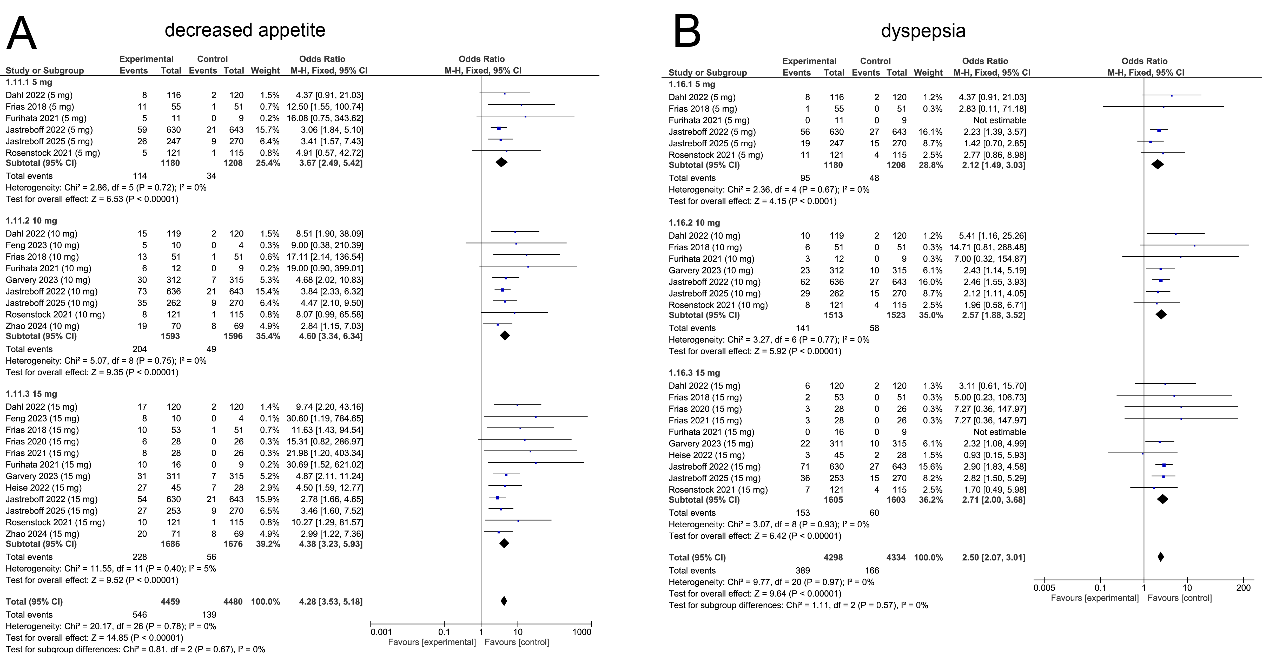


Supplemental Figure S5. Forest plot of decreased appetite (A) and dyspepsia (B).


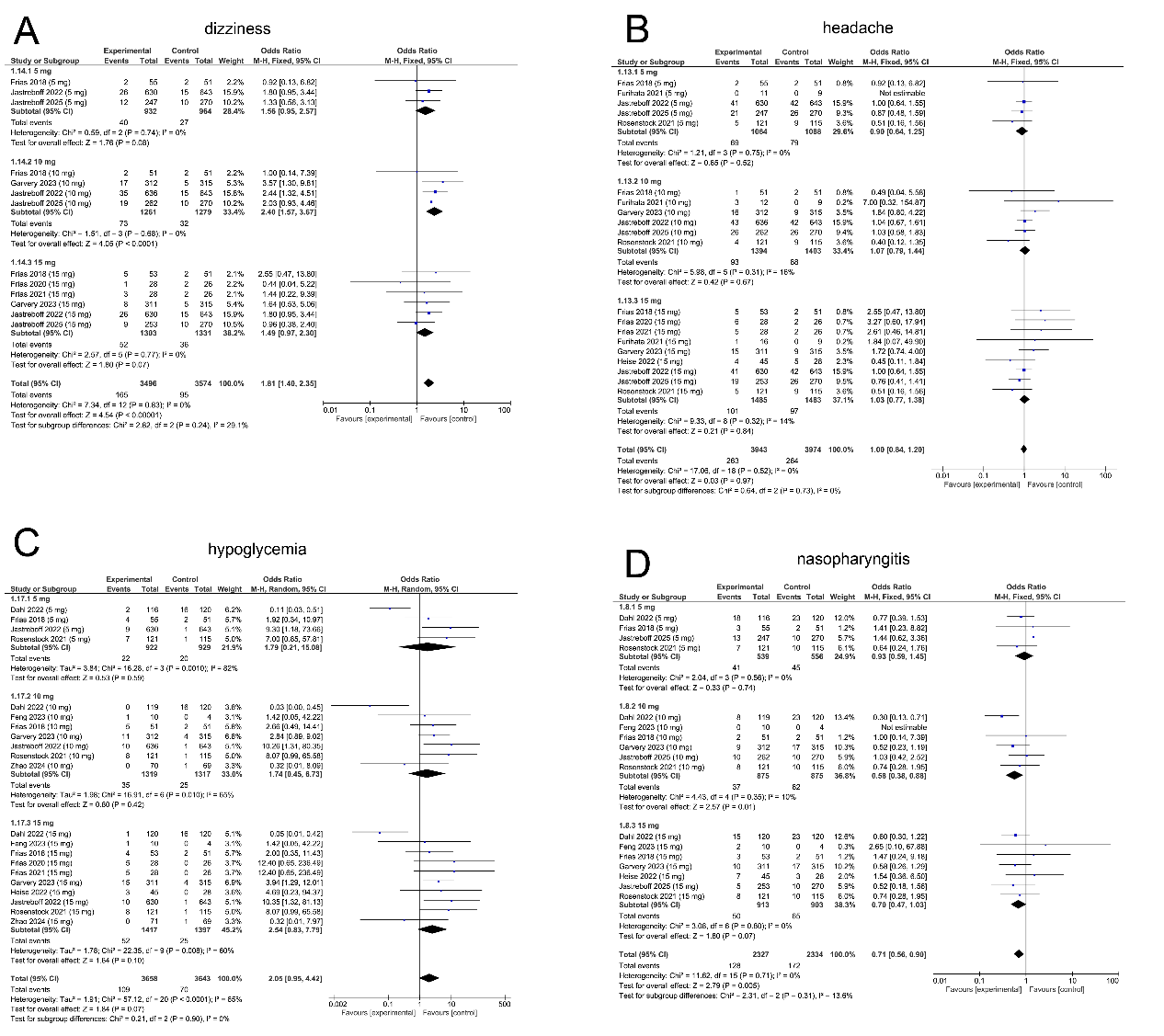


Supplemental Figure S6. Forest plot of dizziness (A), headache (B), hypoglycemia (C) and nasopharyngitis (D).


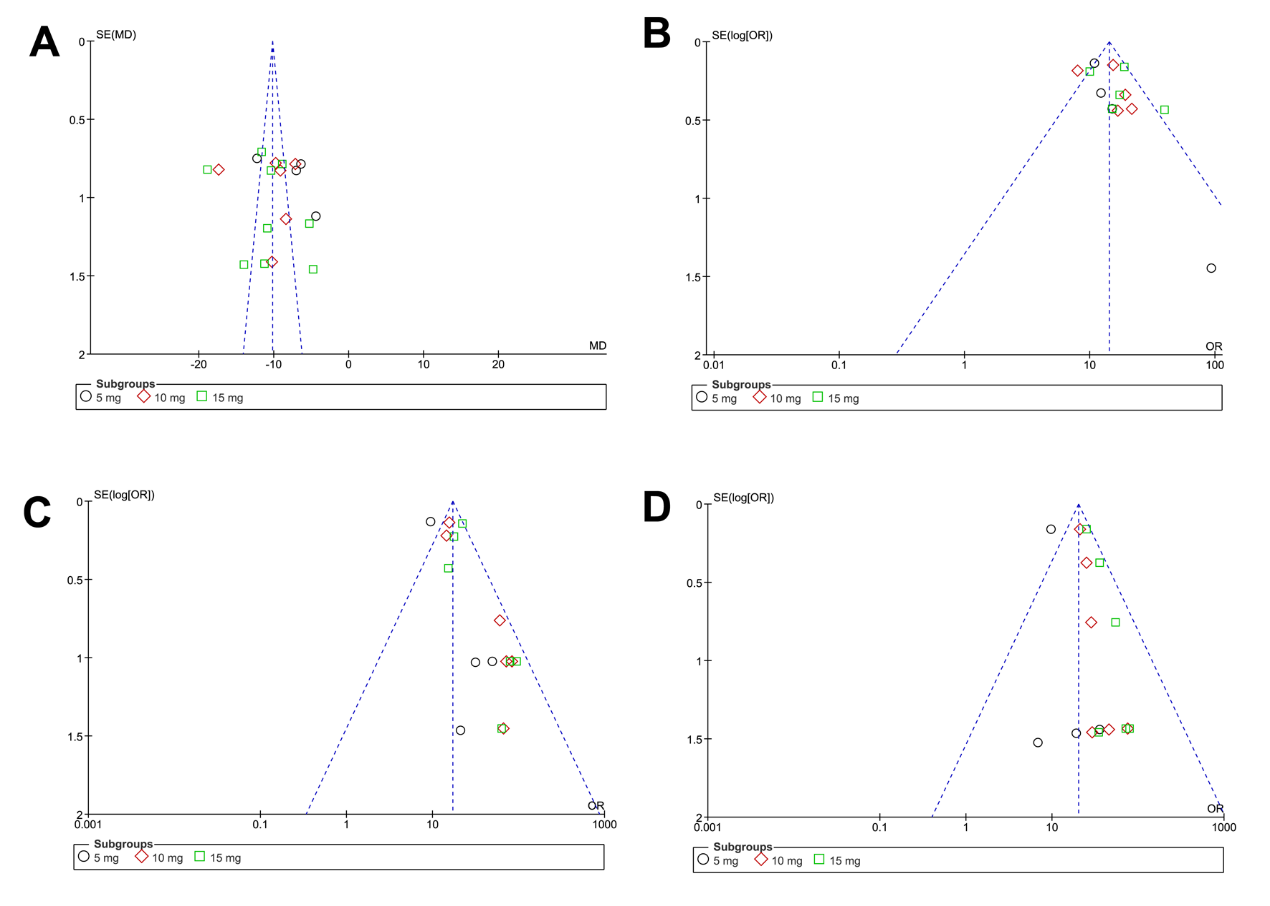


Supplemental Figure S7. Funnel plot of body weight change (A), body weight loss≥5% (B), ≥10% (C) and ≥15% (D).


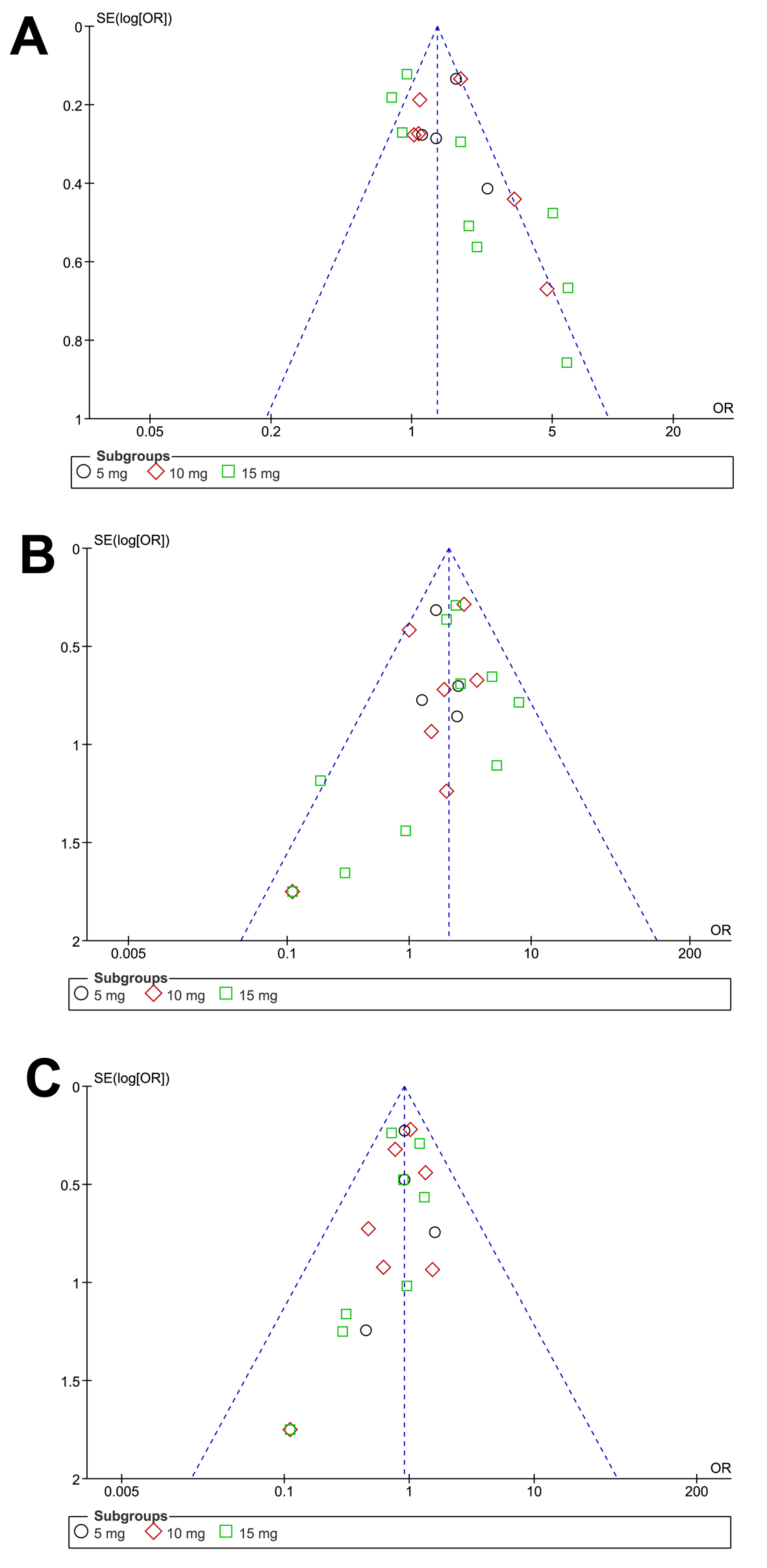


Supplemental Figure S8. Funnel plot of any adverse event (A), serious adverse events (B), and adverse events leading to drug treatment discontinuation (C).


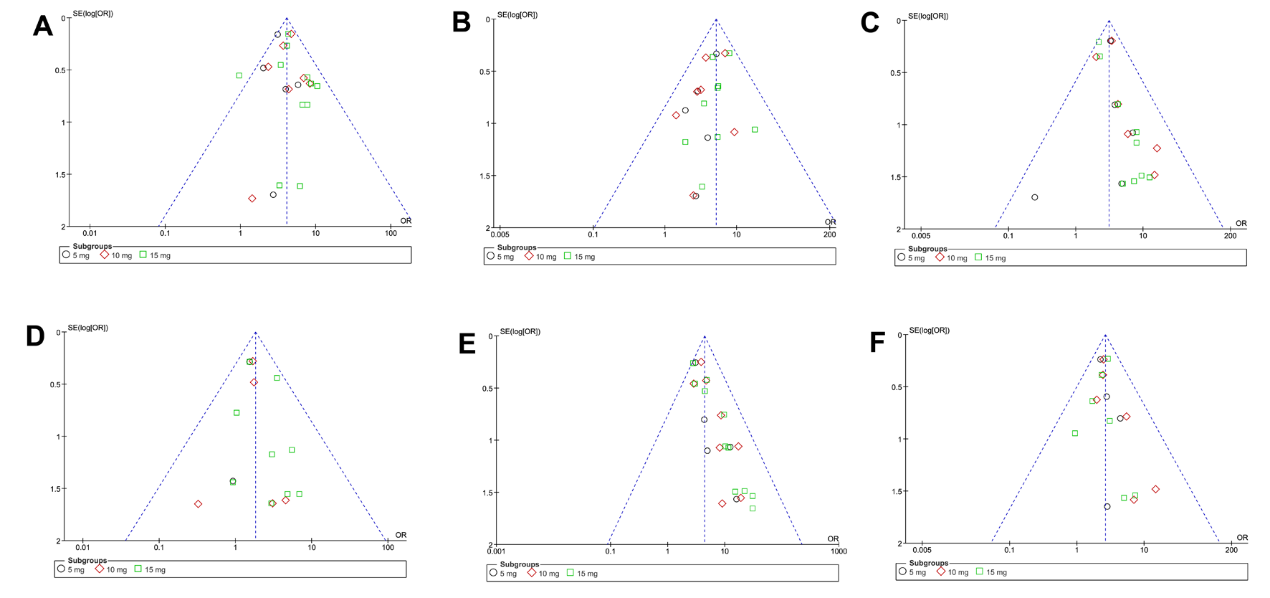


Supplemental Figure S9. Funnel plot of nausea (A), vomiting (B), constipation (C), abdominal pain (D), decreased appetite (E) and dyspepsia (F).


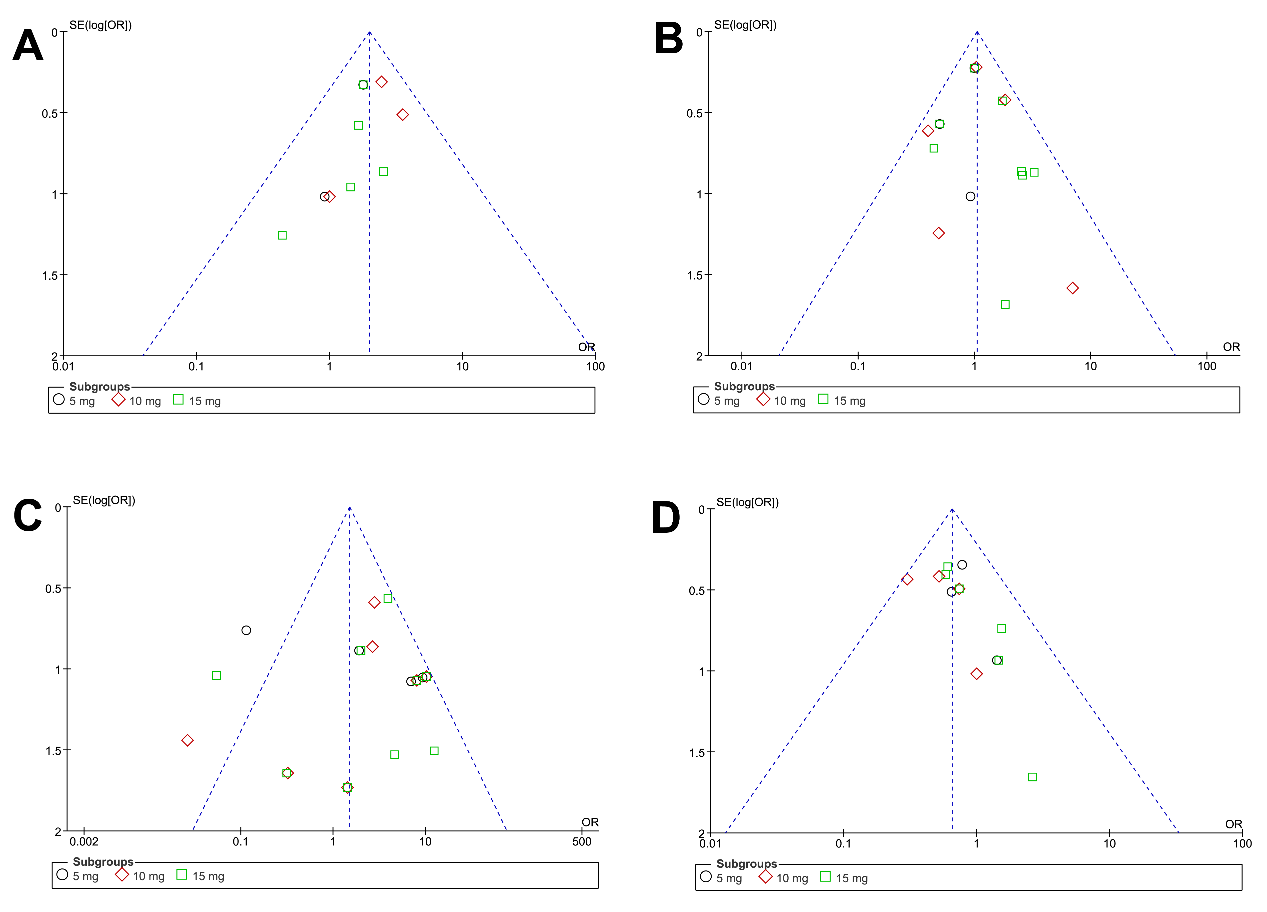


Supplemental Figure S10. Funnel plot of dizziness (A), headache (B), hypoglycemia (C) and nasopharyngitis.
