# Supplementary material for: Efficacy and safety of tirzepatide for weight loss in patients with obesity or type 2 diabetes: a systematic review and meta-analysis
Source: Front Endocrinol (Lausanne). 2025 Jul 17;16:1593134. doi: 10.3389/fendo.2025.1593134 (PMC12310450; doi:10.3389/fendo.2025.1593134)
Supplement: Supplementary file 2 [file Table1.docx]

Supplemental Table S1. Subgroup meta-analysis of body weight changes

|  | Body weight changes (5 mg) | | | Body weight changes (10 mg) | | | Body weight changes (15 mg) | | |
| --- | --- | --- | --- | --- | --- | --- | --- | --- | --- |
|  | Mean difference [95% CI) | P value | Heterogeneity | Mean difference [95% CI) | P value | Heterogeneity | Mean difference [95% CI) | P value | Heterogeneity |
| Diabetes | -6.17 [-7.16, -5.17] | P<0.00001 | Chi² = 3.54, df = 2 (P = 0.17); I² = 43% | -8.57 [-9.41, -7.74] | P<0.00001 | Chi² = 6.07, df = 3 (P = 0.11); I² = 51% | -9.60 [-10.32, -8.89] | P<0.00001 | Chi² = 37.93, df = 6 (P < 0.00001); I² = 84% |
| Non-diabetes | -12.10 [-13.47, -10.72] | P<0.00001 | Chi² = 0.12, df = 1 (P = 0.73); I² = 0% | -15.94 [-17.25, -14.62] | P<0.00001 | Chi² = 22.00, df = 2 (P < 0.0001); I² = 91% | -17.86 [-19.19, -16.54] | P<0.00001 | Chi² = 9.74, df = 2 (P = 0.008); I² = 79% |
|  | 5% weight loss (5 mg) | | | 5% weight loss (10 mg) | | | 5% weight loss (15 mg) | | |
|  | OR [95% CI) | P value | Heterogeneity | OR [95% CI) | P value | Heterogeneity | OR [95% CI) | P value | Heterogeneity |
| Diabetes | 15.70 [9.49, 25.98] | P<0.00001 | Chi² = 2.06, df = 2 (P = 0.36); I² = 3% | 12.20 [9.14, 16.27] | P<0.00001 | Chi² = 13.17, df = 3 (P = 0.004); I² = 77% | 15.01 [11.15, 20.21] | P<0.00001 | Chi² = 12.38, df = 3 (P = 0.006); I² = 76% |
| Non-diabetes | 8.30 [6.56, 10.49] | P<0.00001 | Chi² = 14.19, df = 1 (P = 0.0002); I² = 93% | 10.62 [8.37, 13.46] | P<0.00001 | Chi² = 23.74, df = 2 (P < 0.00001); I² = 92% | 12.13 [9.48, 15.51] | P<0.00001 | Chi² = 26.39, df = 2 (P < 0.00001); I² = 92% |
|  | 10% weight loss (5 mg) | | | 10% weight loss (10 mg) | | | 10% weight loss (15 mg) | | |
|  | OR [95% CI) | P value | Heterogeneity | OR [95% CI) | P value | Heterogeneity | OR [95% CI) | P value | Heterogeneity |
| Diabetes | 35.62 [10.00, 126.86] | P<0.00001 | Chi² = 0.25, df = 2 (P = 0.88); I² = 0% | 21.51 [14.23, 32.51] | P<0.00001 | Chi² = 6.73, df = 3 (P = 0.08); I² = 55% | 25.53 [16.80, 38.79] | P<0.00001 | Chi² = 5.98, df = 3 (P = 0.11); I² = 50% |
| Non-diabetes | 8.61 [6.77, 10.93] | P<0.00001 | Chi² = 2.33, df = 1 (P = 0.13); I² = 57% | 13.88 [10.86, 17.74] | P<0.00001 | Chi² = 9.98, df = 2 (P = 0.007); I² = 80% | 17.02 [13.24, 21.88] | P<0.00001 | Chi² = 10.18, df = 2 (P = 0.006); I² = 80% |
|  | 15% weight loss (5 mg) | | | 15% weight loss (10 mg) | | | 15% weight loss (15 mg) | | |
|  | OR [95% CI) | P value | Heterogeneity | OR [95% CI) | P value | Heterogeneity | OR [95% CI) | P value | Heterogeneity |
| Diabetes | 20.03 [3.87, 103.64] | P<0.00001 | Chi² = 0.65, df = 2 (P = 0.72); I² = 0% | 30.19 [15.33, 59.44] | P<0.00001 | Chi² = 0.71, df = 3 (P = 0.87); I² = 0% | 41.04 [20.76, 81.11] | P<0.00001 | Chi² = 0.54, df = 3 (P = 0.91); I² = 0% |
| Non-diabetes | 9.12 [6.80, 12.23] | P<0.00001 | Chi² = 0.72, df = 1 (P = 0.40); I² = 0% | 18.58 [13.86, 24.90] | P<0.00001 | Chi² = 3.40, df = 2 (P = 0.18); I² = 41% | 22.65 [16.86, 30.44] | P<0.00001 | Chi² = 4.74, df = 2 (P = 0.09); I² = 58% |

Supplemental Table S2. Subgroup meta-analysis of adverse events

|  | Any adverse event (5 mg) | | | Any adverse event (10 mg) | | | Any adverse event (15 mg) | | |
| --- | --- | --- | --- | --- | --- | --- | --- | --- | --- |
|  | OR [95% CI) | P value | Heterogeneity | OR [95% CI) | P value | Heterogeneity | OR [95% CI) | P value | Heterogeneity |
| Diabetes | 1.38 [0.97, 1.96] | P=0.05 | Chi² = 2.30, df = 2 (P = 0.32); I² = 13% | 1.19 [0.92, 1.53] | P=0.0003 | Chi² = 5.72, df = 3 (P = 0.13); I² = 48% | 1.26 [1.00, 1.59] | P<0.00001 | Chi² = 27.15, df = 6 (P = 0.0001); I² = 78% |
| Non-diabetes | 1.53 [1.22, 1.92] | P=0.0003 | Chi² = 1.38, df = 1 (P = 0.24); I² = 28% | 1.75 [1.40, 2.20] | P<0.00001 | Chi² = 2.52, df = 2 (P = 0.28); I² = 21% | 1.05 [0.85, 1.30] | P=0.63 | Chi² = 3.18, df = 2 (P = 0.20); I² = 37% |
|  | Serious adverse events (5 mg) | | | Serious adverse events (10 mg) | | | Serious adverse events (15 mg) | | |
|  | OR [95% CI) | P value | Heterogeneity | OR [95% CI) | P value | Heterogeneity | OR [95% CI) | P value | Heterogeneity |
| Diabetes | 1.00 [0.48, 2.10] | P>0.05 | Chi² = 0.84, df = 2 (P = 0.66); I² = 0% | 0.90 [0.57, 1.44] | P>0.05 | Chi² = 2.95, df = 4 (P = 0.57); I² = 0% | 0.94 [0.60, 1.47] | P>0.05 | Chi² = 3.99, df = 5 (P = 0.55); I² = 0% |
| Non-diabetes | 0.98 [0.70, 1.38] | P>0.05 | Chi² = 0.17, df = 1 (P = 0.68); I² = 0% | 1.06 [0.77, 1.46] | P>0.05 | Chi² = 1.75, df = 2 (P = 0.42); I² = 0% | 0.93 [0.67, 1.29] | P>0.05 | Chi² = 2.12, df = 2 (P = 0.35); I² = 5% |
|  | Adverse event leading to study drug discontinuation (5 mg) | | | Adverse event leading to study drug discontinuation (10 mg) | | | Adverse event leading to study drug discontinuation (15 mg) | | |
|  | OR [95% CI) | P value | Heterogeneity | OR [95% CI) | P value | Heterogeneity | OR [95% CI) | P value | Heterogeneity |
| Diabetes | 2.01 [0.85, 4.78] | P>0.05 | Chi² = 0.49, df = 2 (P = 0.78); I² = 0% | 1.44 [0.82, 2.51] | P>0.05 | Chi² = 4.90, df = 4 (P = 0.30); I² = 18% | 2.17 [1.37, 3.45] | P=0.001 | Chi² = 13.21, df = 7 (P = 0.07); I² = 47% |
| Non-diabetes | 1.46 [0.92, 2.32] | P>0.05 | Chi² = 0.34, df = 1 (P = 0.56); I² = 0% | 2.26 [1.48, 3.43] | P=0.0001 | Chi² = 1.37, df = 2 (P = 0.50); I² = 0% | 2.42 [1.59, 3.67] | P<0.0001 | Chi² = 0.54, df = 2 (P = 0.76); I² = 0% |
|  | Nasopharyngitis (5 mg) | | | Nasopharyngitis (10 mg) | | | Nasopharyngitis (15 mg) | | |
|  | OR [95% CI) | P value | Heterogeneity | OR [95% CI) | P value | Heterogeneity | OR [95% CI) | P value | Heterogeneity |
| Diabetes | 0.77 [0.45, 1.32] | P>0.05 | Heterogeneity: Chi² = 0.54, df = 2 (P = 0.76); I² = 0% | 0.49 [0.30, 0.79] | P=0.004 | Heterogeneity: Chi² = 2.44, df = 3 (P = 0.49); I² = 0% | 0.73 [0.48, 1.11] | P>0.05 | Heterogeneity: Chi² = 2.78, df = 5 (P = 0.73); I² = 0% |
| Non-diabetes | 1.44 [0.62, 3.36] | P>0.05 | N/A | 1.03 [0.42, 2.52] | P>0.05 | N/A | 0.52 [0.18, 1.56] | P>0.05 | N/A |
|  | Nausea (5 mg) | | | Nausea (10 mg) | | | Nausea (15 mg) | | |
|  | OR [95% CI) | P value | Heterogeneity | OR [95% CI) | P value | Heterogeneity | OR [95% CI) | P value | Heterogeneity |
| Diabetes | 3.28 [1.73, 6.19] | P=0.0003 | Chi² = 1.88, df = 3 (P = 0.60); I² = 0% | 3.84 [2.56, 5.75] | P<0.0001 | Heterogeneity: Chi² = 2.97, df = 4 (P = 0.56); I² = 0% | 4.32 [3.05, 6.11] | P<0.0001 | Chi² = 11.38, df = 8 (P = 0.18); I² = 30% |
| Non-diabetes | 2.10 [1.64, 2.69] | P<0.0001 | Chi² = 16.21, df = 1 (P < 0.0001); I² = 94% | 3.32 [2.64, 4.18] | P<0.0001 | Chi² = 19.88, df = 2 (P < 0.0001); I² = 90% | 3.08 [2.44, 3.89] | P<0.0001 | Chi² = 18.82, df = 2 (P < 0.0001); I² = 89% |
|  | Vomiting (5 mg) | | | Vomiting (10 mg) | | | Vomiting (15 mg) | | |
|  | OR [95% CI) | P value | Heterogeneity | OR [95% CI) | P value | Heterogeneity | OR [95% CI) | P value | Heterogeneity |
| Diabetes | 2.73 [1.09, 6.82] | P=0.03 | Chi² = 0.26, df = 3 (P = 0.97); I² = 0% | 3.60 [2.05, 6.30] | P<0.0001 | Chi² = 1.85, df = 4 (P = 0.76); I² = 0% | 5.08 [3.08, 8.37] | P<0.0001 | Chi² = 2.49, df = 7 (P = 0.93); I² = 0% |
| Non-diabetes | 5.27 [2.99, 9.27] | P<0.0001 | Chi² = 0.01, df = 1 (P = 0.91); I² = 0% | 6.44 [3.87, 10.71] | P<0.0001 | Chi² = 1.56, df = 2 (P = 0.46); I² = 0% | 8.24 [4.98, 13.63] | P<0.0001 | Chi² = 0.71, df = 2 (P = 0.70); I² = 0% |
|  | Decreased appetite (5 mg) | | | Decreased appetite (10 mg) | | | Decreased appetite (15 mg) | | |
|  | OR [95% CI) | P value | Heterogeneity | OR [95% CI) | P value | Heterogeneity | OR [95% CI) | P value | Heterogeneity |
| Diabetes | 7.10 [2.59, 19.44] | P=0.0001 | Chi² = 1.03, df = 3 (P = 0.79); I² = 0% | 7.06 [3.80, 13.13] | P<0.0001 | Chi² = 2.12, df = 5 (P = 0.83); I² = 0% | 7.55 [4.53, 12.58] | P<0.0001 | Chi² = 4.65, df = 8 (P = 0.79); I² = 0% |
| Non-diabetes | 3.16 [2.06, 4.85] | P<0.0001 | Chi² = 0.05, df = 1 (P = 0.82); I² = 0% | 3.81 [2.61, 5.55] | P<0.0001 | Chi² = 0.58, df = 2 (P = 0.75); I² = 0% | 2.98 [2.02, 4.39] | P<0.0001 | Chi² = 0.22, df = 2 (P = 0.90); I² = 0% |
|  | Constipation (5 mg) | | | Constipation (10 mg) | | | Constipation (15 mg) | | |
|  | OR [95% CI) | P value | Heterogeneity | OR [95% CI) | P value | Heterogeneity | OR [95% CI) | P value | Heterogeneity |
| Diabetes | 3.39 [1.23, 9.31] | P=0.0w | Chi² = 2.92, df = 3 (P = 0.40); I² = 0% | 3.24 [1.86, 5.64] | P<0.0001 | Chi² = 4.96, df = 4 (P = 0.29); I² = 19% | 3.05 [2.22, 4.19] | P<0.0001 | Chi² = 4.17, df = 7 (P = 0.76); I² = 0% |
| Non-diabetes | 3.05 [2.22, 4.19] | P<0.0001 | Chi² = 0.54, df = 1 (P = 0.46); I² = 0% | 3.30 [2.41, 4.51] | P<0.0001 | Chi² = 0.05, df = 1 (P = 0.82); I² = 0% | 2.16 [1.55, 3.00] | P<0.0001 | Heterogeneity: Chi² = 0.01, df = 1 (P = 0.93); I² = 0% |
|  | Headache (5 mg) | | | Headache (10 mg) | | | Headache (15 mg) | | |
|  | OR [95% CI) | P value | Heterogeneity | OR [95% CI) | P value | Heterogeneity | OR [95% CI) | P value | Heterogeneity |
| Diabetes | 0.58 [0.22, 1.55] | P>0.05 | Chi² = 0.26, df = 1 (P = 0.61); I² = 0% | 1.17 [0.64, 2.12] | P>0.05 | Chi² = 5.91, df = 3 (P = 0.12); I² = 49% | 1.30 [0.80, 2.11] | P>0.05 | Chi² = 7.70, df = 6 (P = 0.26); I² = 22% |
| Non-diabetes | 0.95 [0.66, 1.36] | P>0.05 | Chi² = 0.12, df = 1 (P = 0.73); I² = 0% | 1.04 [0.73, 1.47] | P>0.05 | Chi² = 0.00, df = 1 (P = 0.99); I² = 0% | 0.91 [0.63, 1.30] | P>0.05 | Chi² = 0.48, df = 1 (P = 0.49); I² = 0% |
|  | Dizziness (5 mg) | | | Dizziness (10 mg) | | | Dizziness (15 mg) | | |
|  | OR [95% CI) | P value | Heterogeneity | OR [95% CI) | P value | Heterogeneity | OR [95% CI) | P value | Heterogeneity |
| Diabetes | 0.92 [0.13, 6.82] | P>0.05 | N/A | 2.83 [1.17, 6.81] | P=0.02 | Chi² = 1.24, df = 1 (P = 0.26); I² = 20% | 1.54 [0.71, 3.34] | P>0.05 | Chi² = 1.34, df = 3 (P = 0.72); I² = 0% |
| Non-diabetes | 1.62 [0.97, 2.70] | P>0.05 | Chi² = 0.31, df = 1 (P = 0.58); I² = 0% | 2.28 [1.40, 3.70] | P=0.0008 | Chi² = 0.13, df = 1 (P = 0.72); I² = 0% | 1.47 [0.87, 2.47] | P>0.05 | Chi² = 1.22, df = 1 (P = 0.27); I² = 18% |
|  | Abdominal pain (5 mg) | | | Abdominal pain (10 mg) | | | Abdominal pain (15 mg) | | |
|  | OR [95% CI) | P value | Heterogeneity | OR [95% CI) | P value | Heterogeneity | OR [95% CI) | P value | Heterogeneity |
| Diabetes | 0.93 [0.06, 15.20] | P>0.05 | N/A | 1.73 [0.77, 3.91] | P>0.05 | Chi² = 1.50, df = 3 (P = 0.68); I² = 0% | 2.97 [1.60, 5.49] | P=0.0005 | Chi² = 3.31, df = 6 (P = 0.77); I² = 0% |
| Non-diabetes | 1.75 [1.10, 2.78] | P=0.02 | Chi² = 0.61, df = 1 (P = 0.43); I² = 0% | 1.77 [1.12, 2.81] | P=0.01 | Chi² = 0.13, df = 1 (P = 0.72); I² = 0% | 1.57 [0.98, 2.50] | P>0.05 | Chi² = 0.16, df = 2 (P = 0.93); I² = 0% |
|  | Dyspepsia (5 mg) | | | Dyspepsia (10 mg) | | | Dyspepsia (15 mg) | | |
|  | OR [95% CI) | P value | Heterogeneity | OR [95% CI) | P value | Heterogeneity | OR [95% CI) | P value | Heterogeneity |
| Diabetes | 3.26 [1.33, 8.02] | P=0.01 | Chi² = 0.21, df = 2 (P = 0.90); I² = 0% | 3.12 [1.78, 5.47] | P<0.0001 | Chi² = 2.81, df = 4 (P = 0.59); I² = 0% | 2.41 [1.41, 4.13] | P=0.001 | Chi² = 2.66, df = 6 (P = 0.85); I² = 0% |
| Non-diabetes | 1.94 [1.31, 2.87] | P=0.0009 | Chi² = 1.10, df = 1 (P = 0.29); I² = 9% | 2.34 [1.60, 3.42] | P<0.0001 | Chi² = 0.14, df = 1 (P = 0.71); I² = 0% | 2.87 [1.98, 4.16] | P<0.0001 | Chi² = 0.00, df = 1 (P = 0.95); I² = 0% |
|  | Hypoglycaemia (5 mg) | | | Hypoglycaemia (10 mg) | | | Hypoglycaemia (15 mg) | | |
|  | OR [95% CI) | P value | Heterogeneity | OR [95% CI) | P value | Heterogeneity | OR [95% CI) | P value | Heterogeneity |
| Diabetes | 1.06 [0.09, 12.36] | P>0.05 | Tau² = 3.87; Chi² = 11.55, df = 2 (P = 0.003); I² = 83% | 1.47 [0.27, 8.07] | P>0.05 | Chi² = 14.21, df = 4 (P = 0.007); I² = 72% | 2.53 [0.70, 9.19] | P>0.05 | Chi² = 19.29, df = 7 (P = 0.007); I² = 64% |
| Non-diabetes | 9.30 [1.18, 73.66] | P=0.03 | N/A | 2.29 [0.08, 66.39] | P>0.05 | Chi² = 3.16, df = 1 (P = 0.08); I² = 68% | 2.28 [0.08, 67.72] | P>0.05 | Chi² = 3.20, df = 1 (P = 0.07); I² = 69% |
